# Supplementary material for: Trypanosoma cruzi cell atlas as a single-cell resource for understanding parasite population heterogeneity and differentiation
Source: Nat Commun. 2026 May 25;17:6801. doi: 10.1038/s41467-026-73098-w (PMC13385397; doi:10.1038/s41467-026-73098-w)
Supplement: Supplementary file 1 — Supplementary Information [file 41467_2026_73098_MOESM1_ESM.pdf]

Supplementary figures and tables

Trypanosoma cruzi cell atlas as a single-cell resource for understanding parasite population heterogeneity and differentiation

Ross F. Laidlaw[1,5%], Marta García-Sánchez[2%], Juliana Da Silva Pacheco[2], Luciana S. Paradela[2], Thomas D. Otto[1,3,4*] and Manu De Rycker[2*]

Affiliations:

1: School of Infection and Immunity, University of Glasgow, Glasgow, UK

2: Drug Discovery Unit, Faculty of Life Sciences, University of Dundee, Dundee, UK

3: Current Address: Bernhard Nocht Institute for Tropical Medicine, Hamburg, Germany

4: Current Address: University of Hamburg, Germany

5: Current Address: Leiden University Medical Centre, The Netherlands

% : contributed equally

* : corresponding authors:

Thomas Otto thomas.otto@bnitm.de; Manu De Rycker M.DeRycker@dundee.ac.uk

Fig. S1. Sampling and library construction.

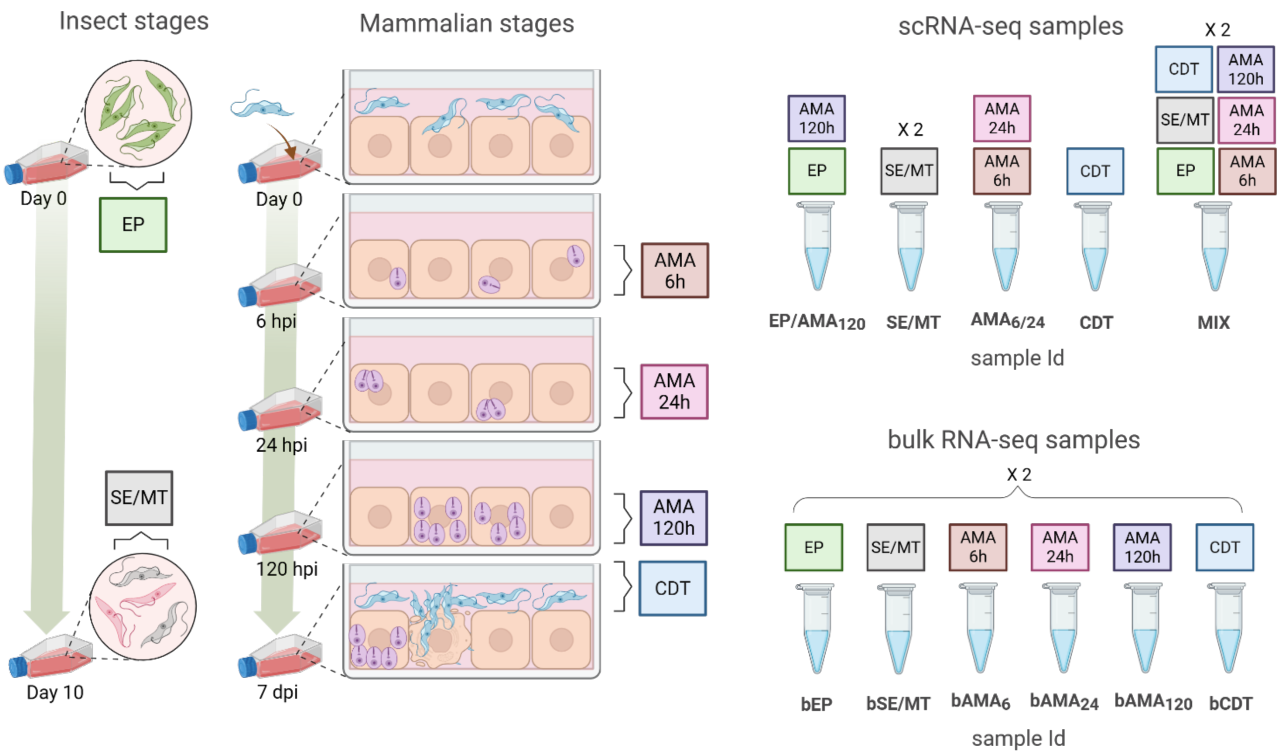

Samples collected from *in vitro* cultures: EP: epimastigotes, collected during the exponential growth phase, SE/MT: a mixture of stationary growth phase epimastigotes and metacyclic trypomastigotes, CDT: cell-derived trypomastigotes, and AMA: amastigotes isolated from Vero-infected cells at 6, 24, and 120 hours post infection (AMA6h, AMA24h and AMA120h respectively). Representative microscopy images for the samples are shown in Fig. S2. Individual libraries were constructed for SE/MT (two biological replicates) and CDT from single-cell suspensions. Two more libraries were prepared by combining AMA6h with AMA24h (AMA6/24), and EP with AMA120h (EP/AMA120) in equal proportion. A second biological replicate was generated by pooling equal numbers of EP, SE/MT, CDT, AMA6h, AMA24h and AMA120h in a single-cell suspension (MIX). Two libraries (technical replicates) were sequenced for MIX. Additional data for sample preparation can be found in Supplementary Table 2. Created in BioRender. Garcia Sanchez, M. (2026) https://BioRender.com/h07k595

Fig. S2: Microscopic images of *in vitro* cultures of all the *T. cruzi* stages used for sample collection.

A

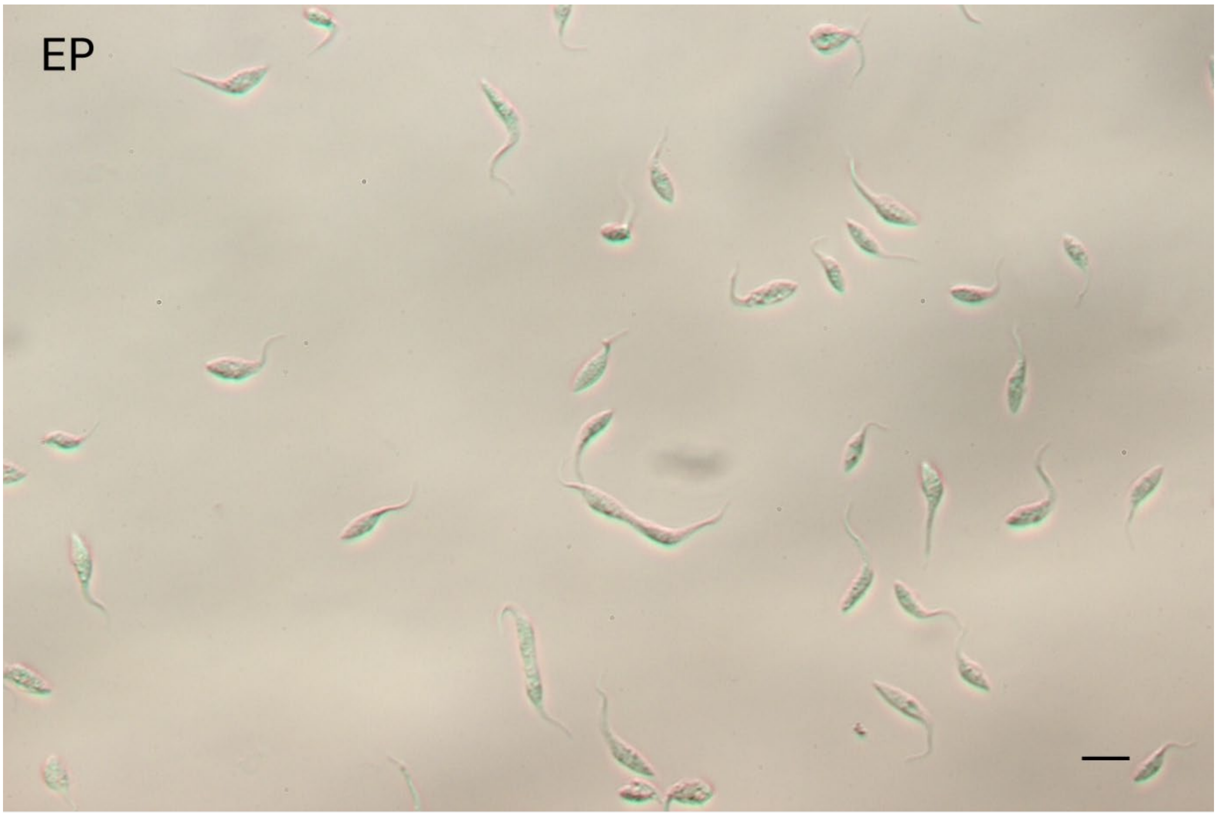

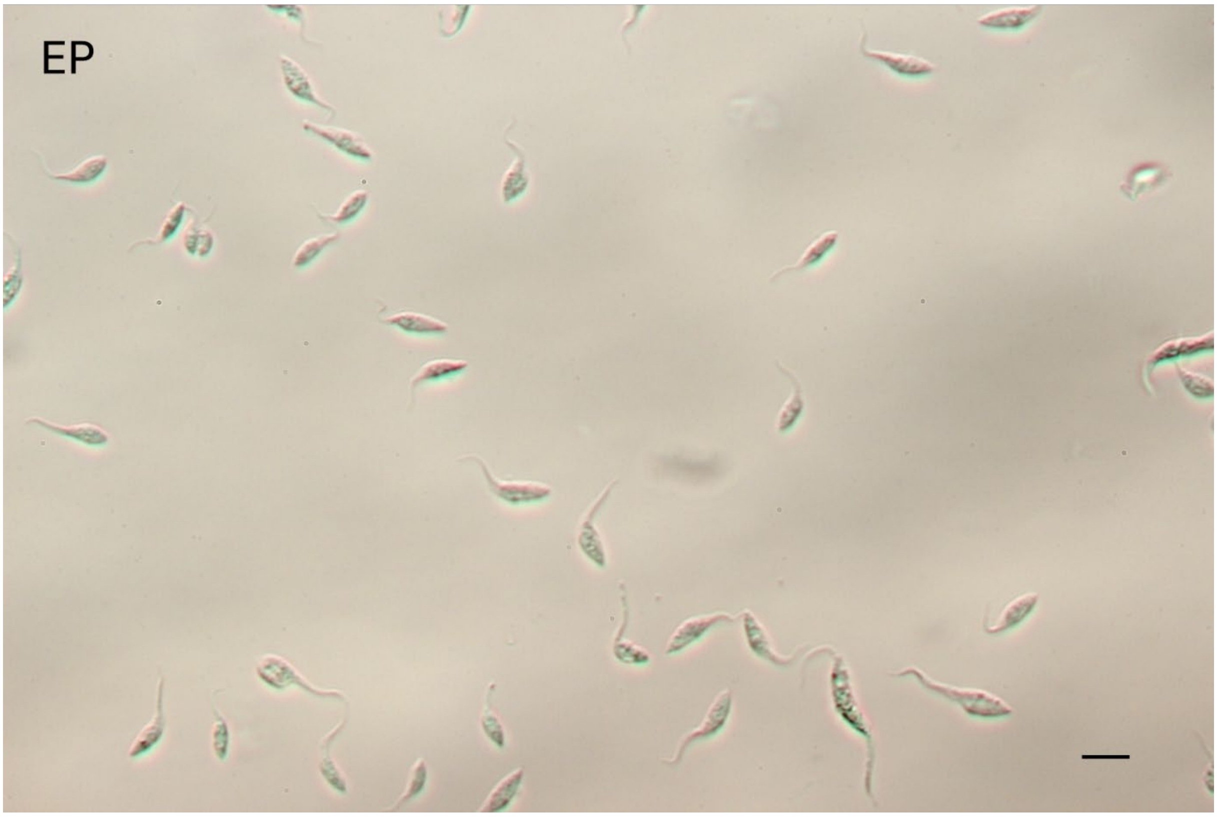

B

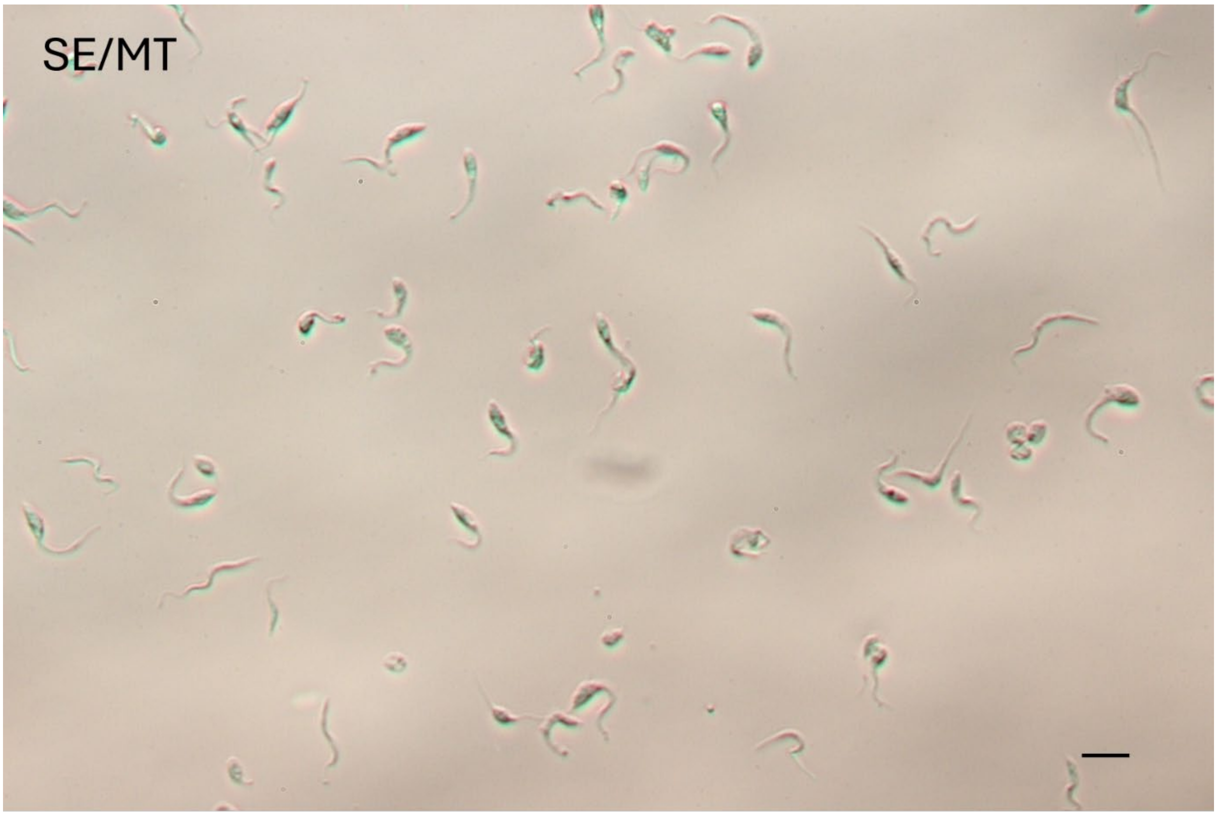

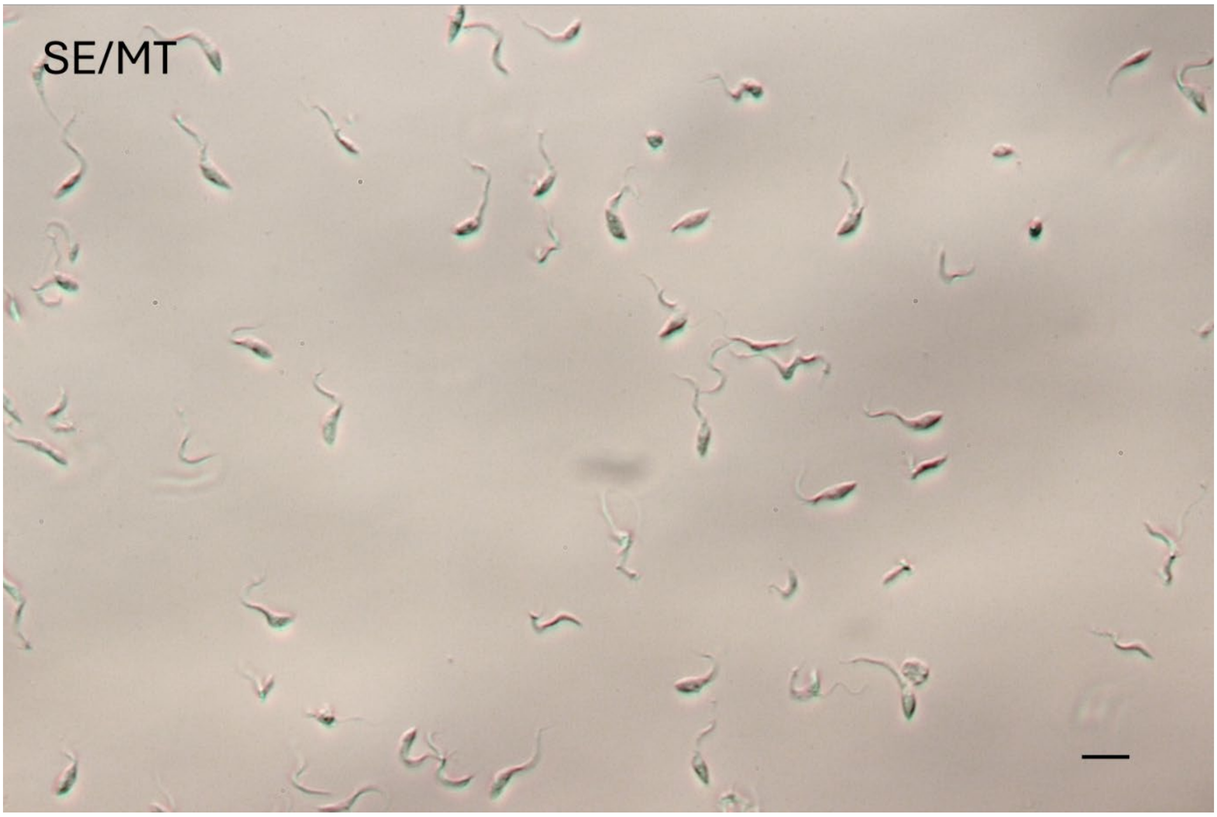

C

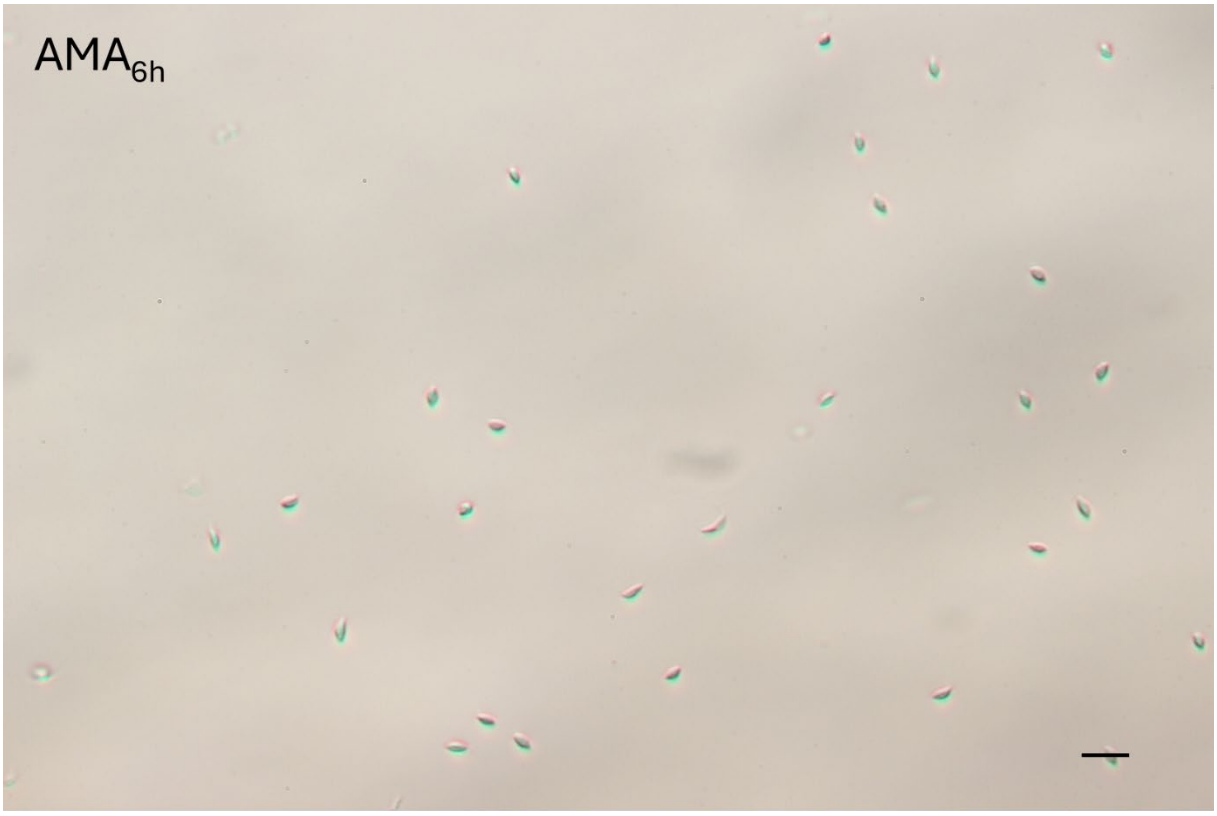

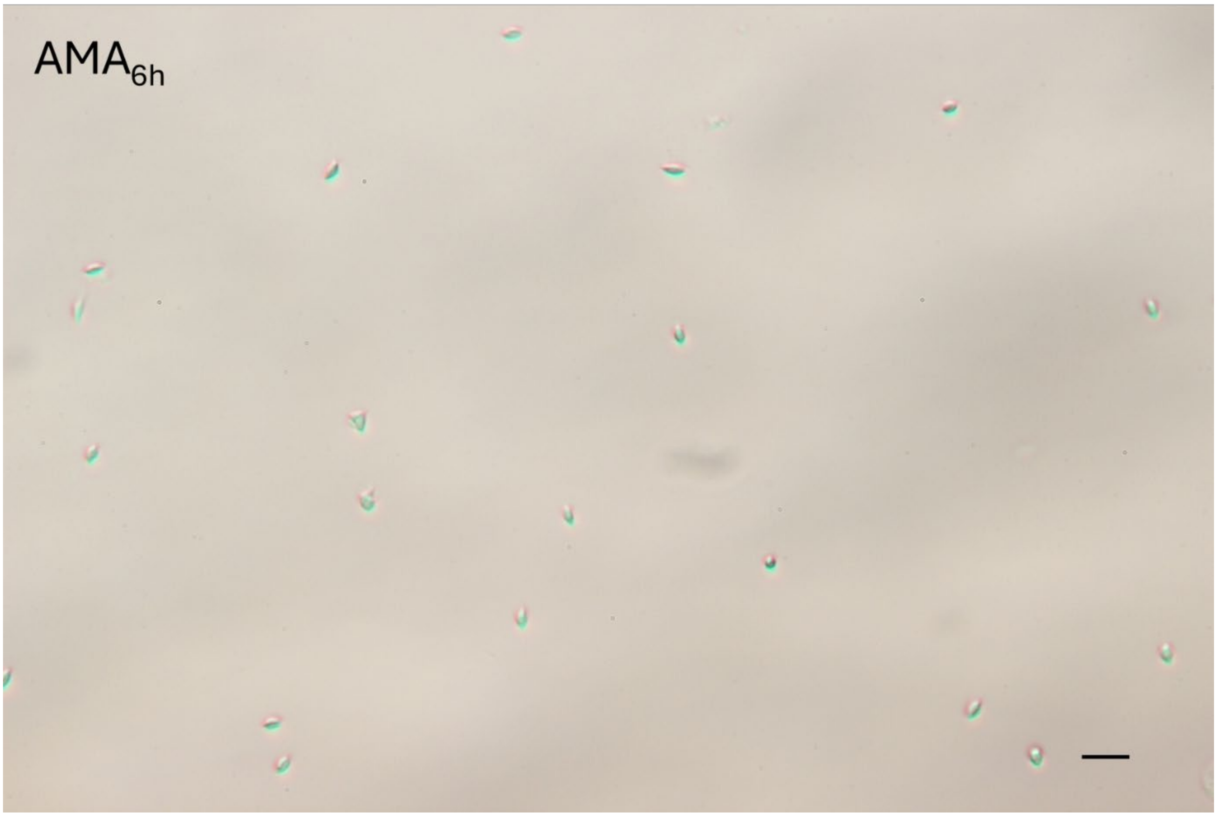

D

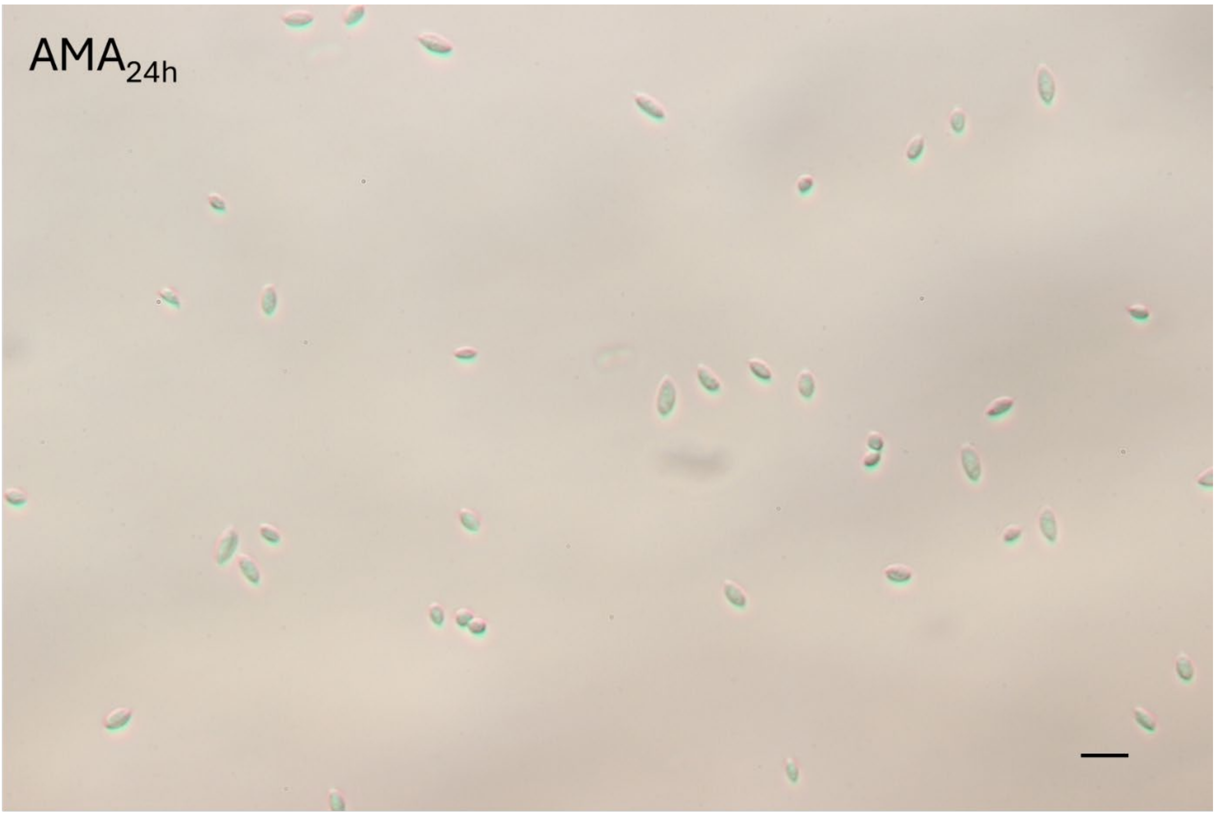

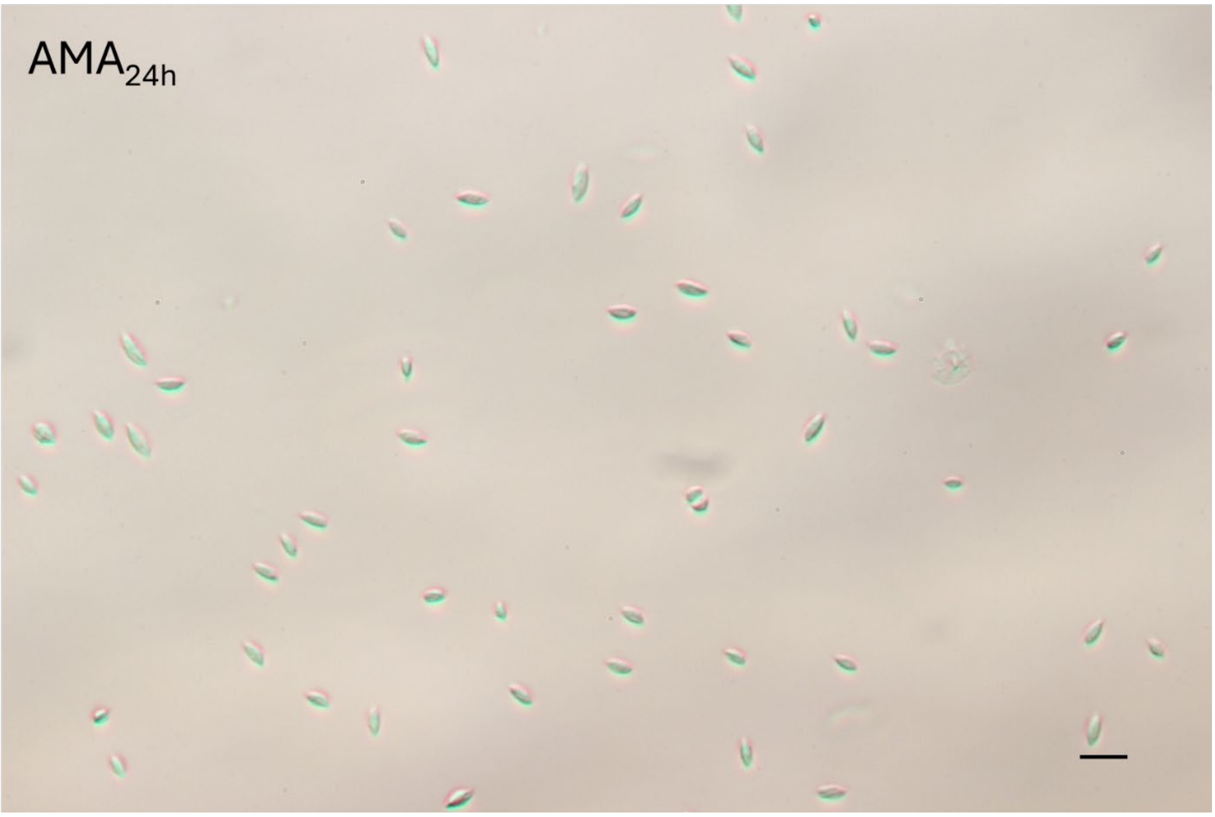

E

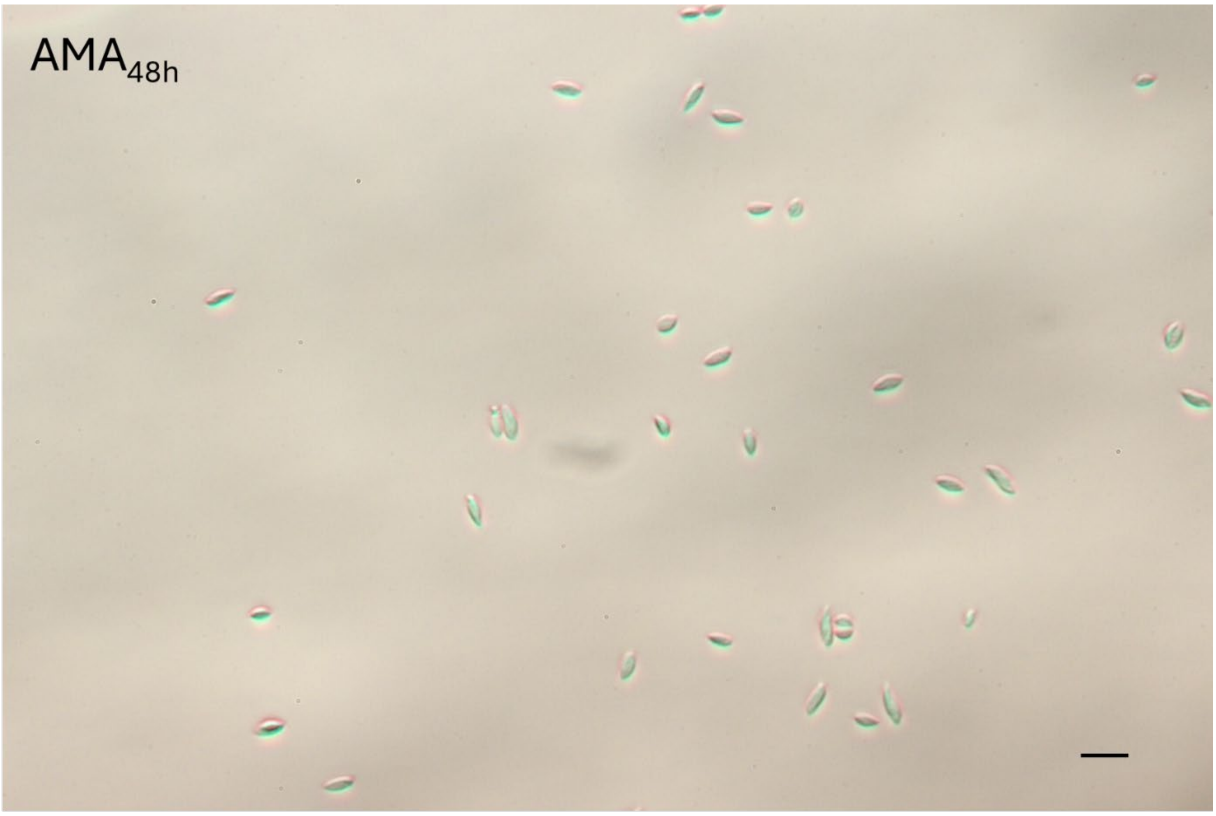

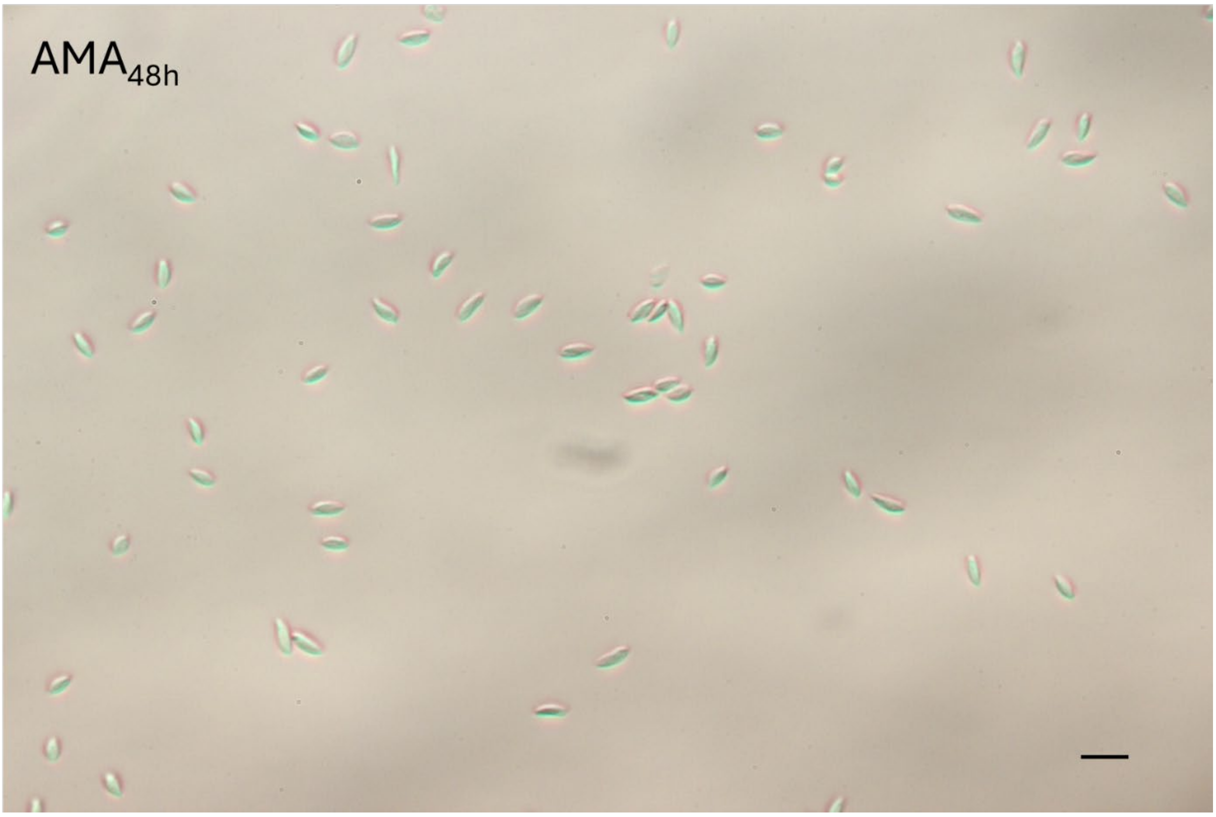

F

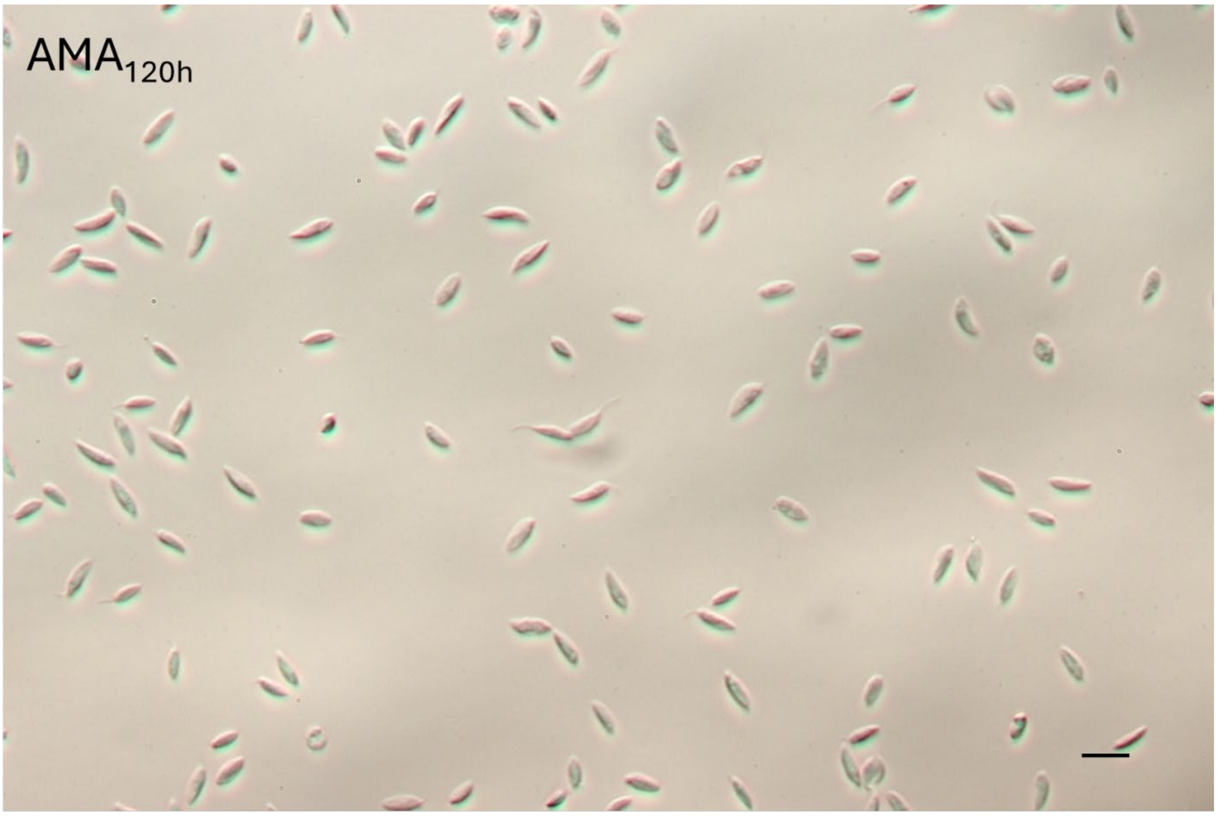

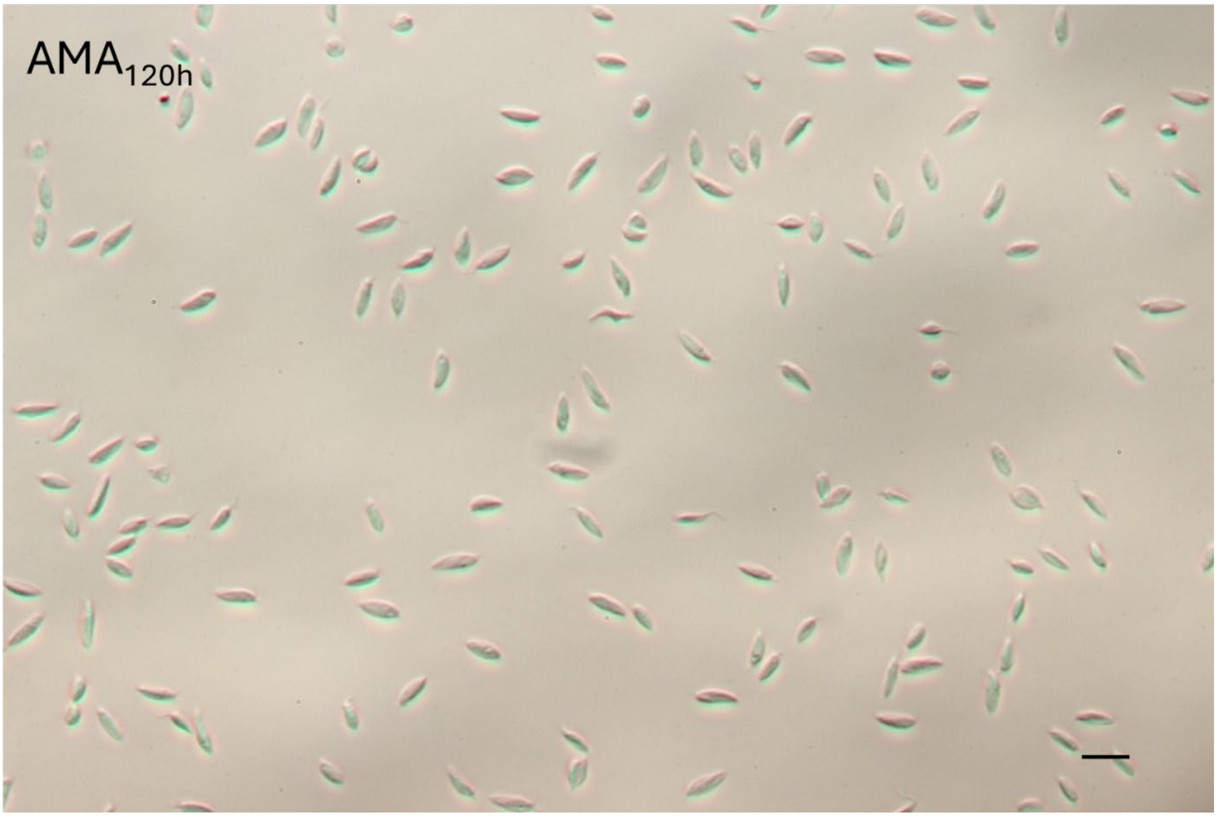

G

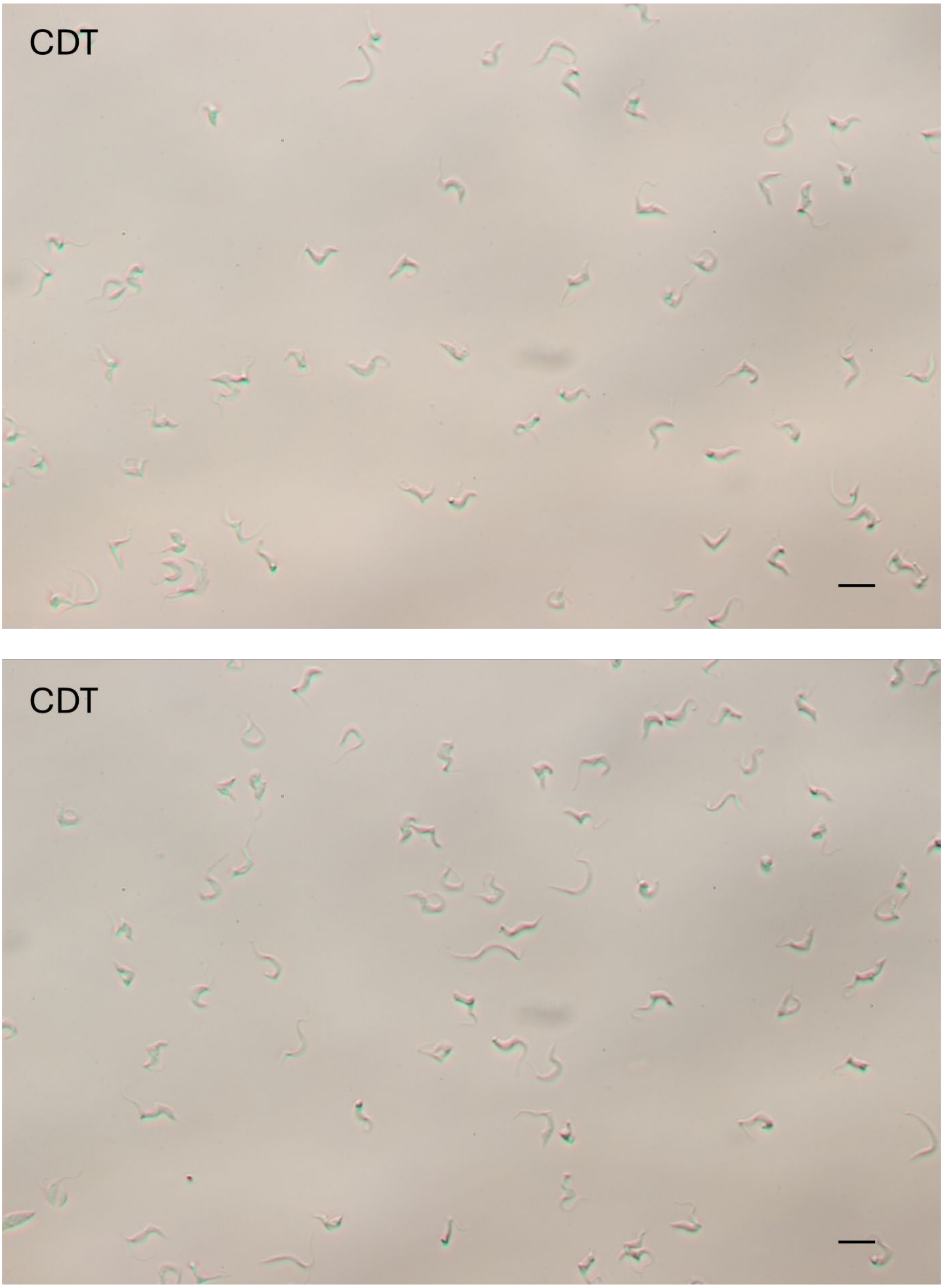

(A) EP: epimastigotes; (B) SE/MT: Mixture of stationary growth phase epimastigotes and metacyclic trypomastigotes obtained by nutritional starvation of epimastigotes; (C) AMA_{6h}: amastigotes isolated from

Vero-infected cells at 6 hpi; (D) AMA_{24h}: amastigotes isolated at 24 hpi; (E) AMA_{48h}: amastigotes isolated at 48 hpi; (F) AMA_{120h}: amastigotes isolated at 120 hpi; (G) CDT: cell-derived trypomastigotes. All cultures were fixed with formaldehyde prior to imaging. Images were acquired using the 40× objective on a Zeiss Axiovert 40 CFL microscope equipped with a Canon EOS 600D camera. Scale bars for all panels: 10 µm.

Fig. S3. Flow cytometry profiles showing the percentage of viable cells for all the samples collected

A

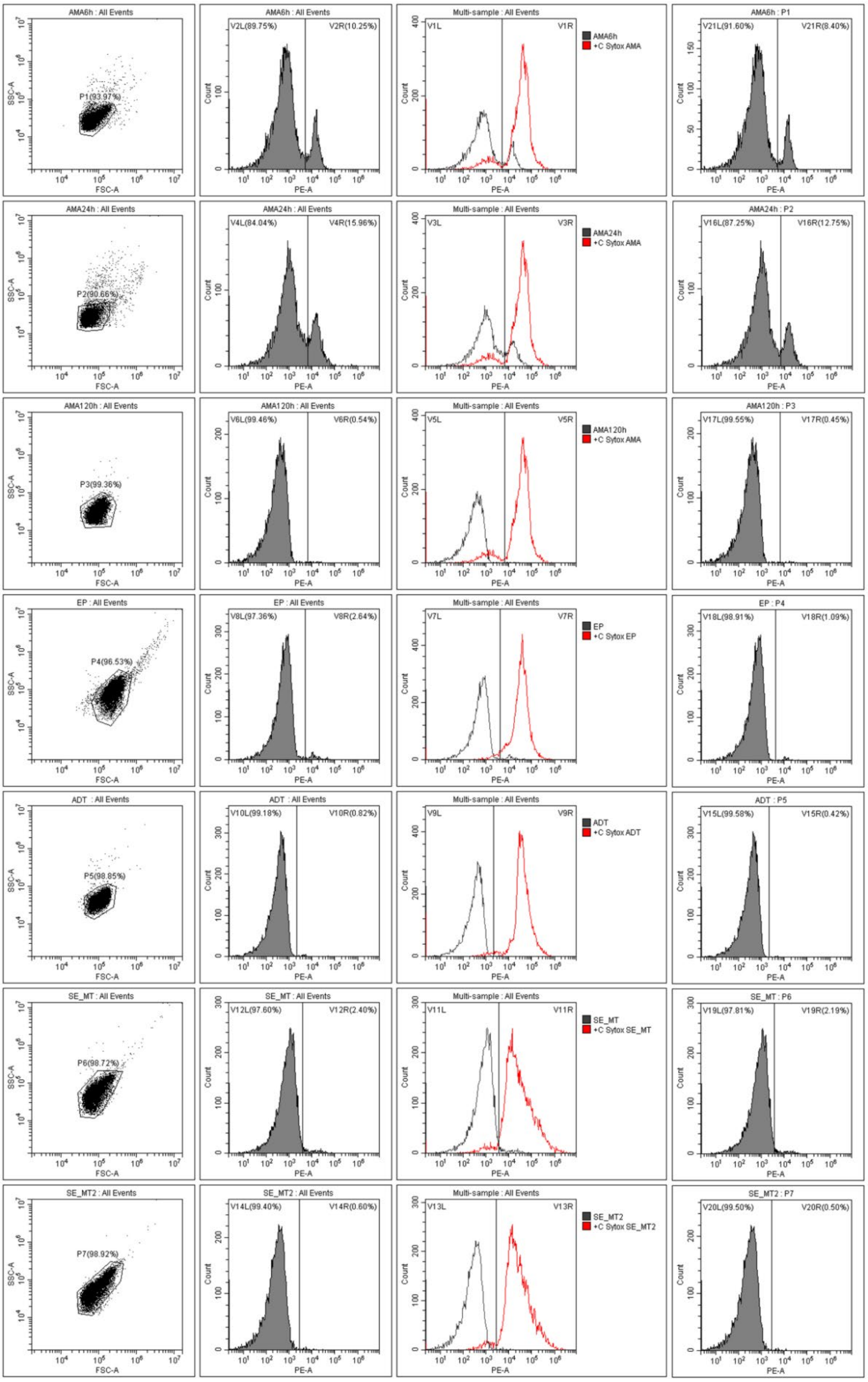

B

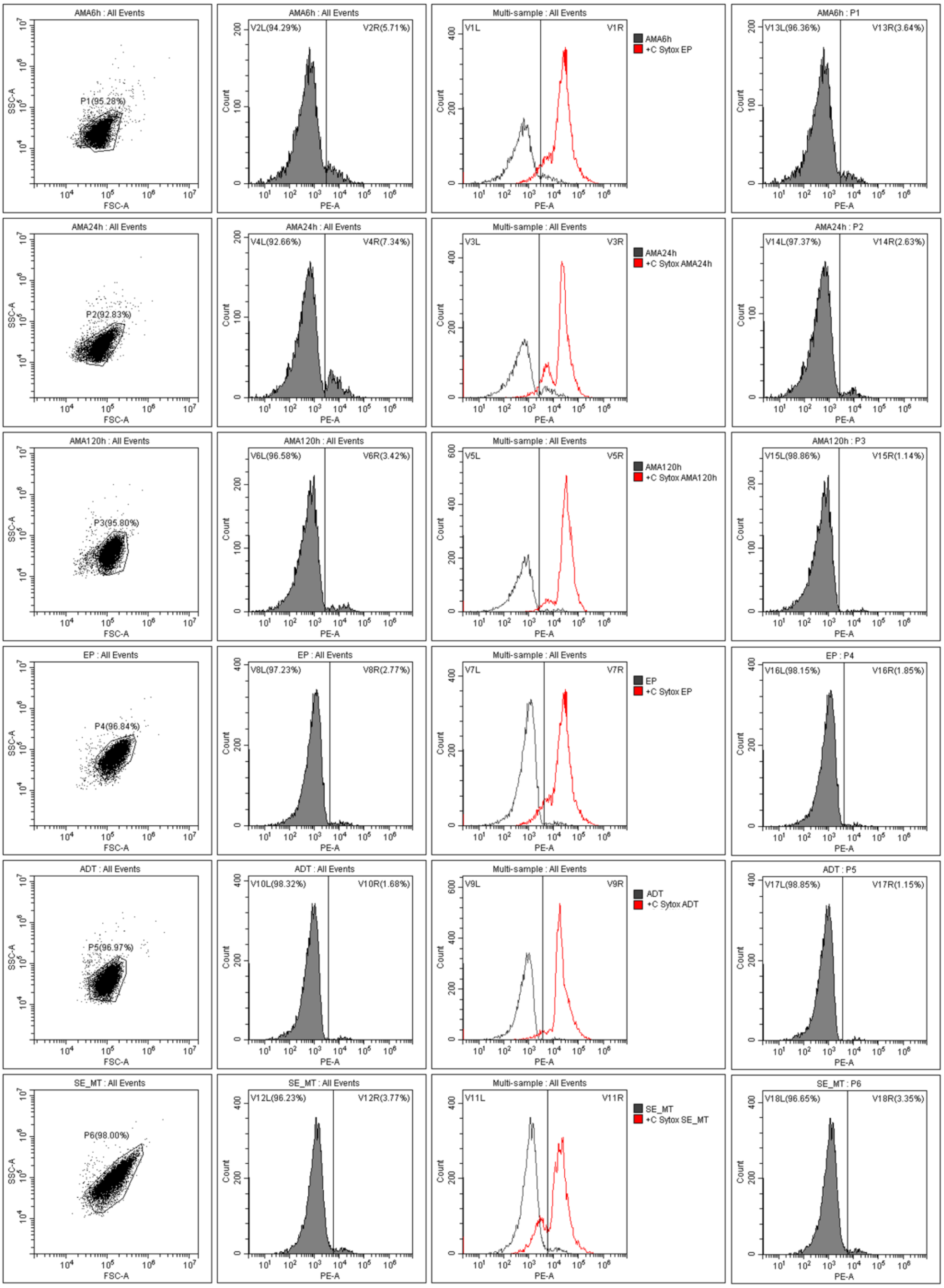

The profiles shown in panel **(A)** correspond to the parasites used for the scRNA-seq samples AMA6/24, EP/AMA120 and SE/MT and the first biological replicate of bulk RNA-seq samples. Those in panel **(B)** were used for the scRNA-seq samples MIX and the second biological replicate of bulk RNA-seq samples.

AMA: amastigotes collected at 6, 24, and 120 hpi (AMA6h, AMA12h and AMA120h respectively); EP: epimastigotes; ADT: cell-derived trypomastigotes; SE/MT: mixture of stationary phase epimastigotes and metacyclic trypomastigotes obtained by nutritional starvation of EP; C+ Sytox (red line): parasites from the different life cycle stages killed with three cycles of freezing and thawing, used as positive control for SYTOX AADvanced staining. The fourth column shows the profiles of the cells gated in the first column to discard cell debris and clumps.

Fig.S4. Frequency of predicted doublets per cluster per batch

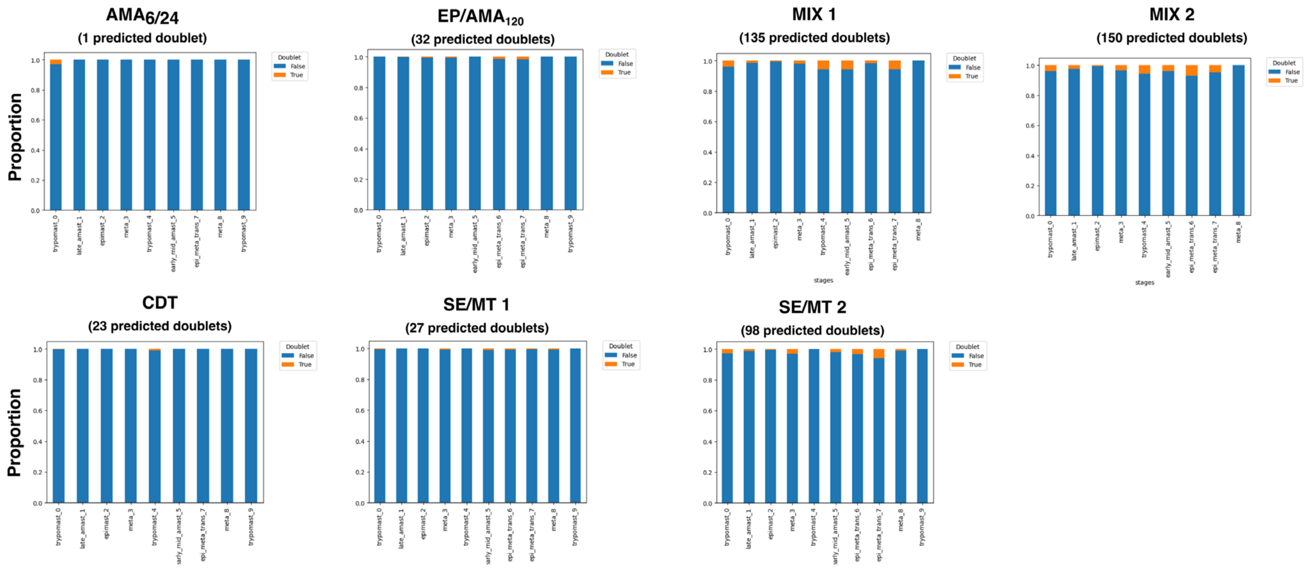

Barplots for each scRNA-seq batch, showing the proportion of Scrublet predicted doublets across all the quality-controlled atlas cells, grouped by the atlas clusters. The number of Scrublet predicted doublets per batch is also reported underneath the batch name.

Fig. S5 Confusion matrices of scPoli lifecycle stage prediction results

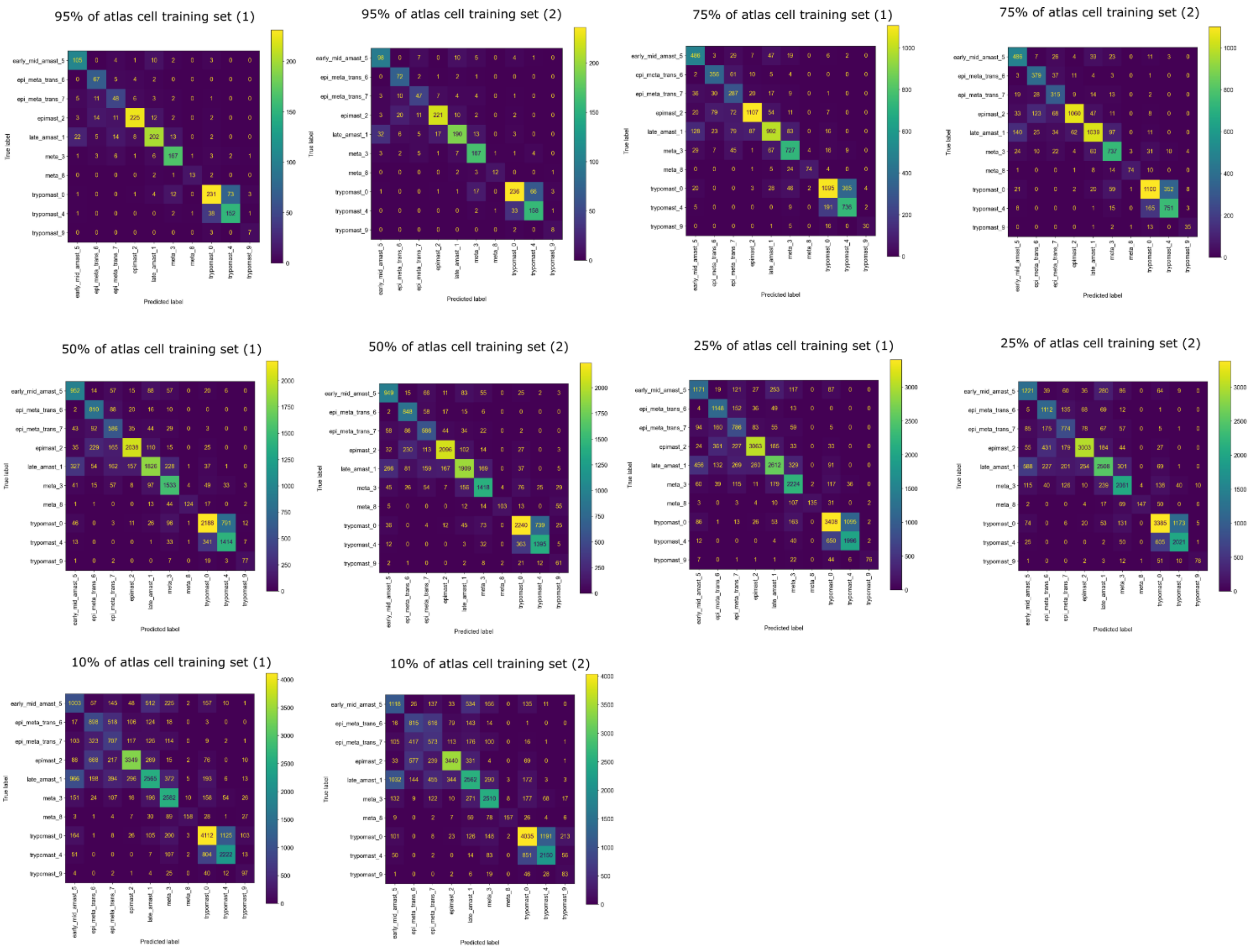

Confusion matrices showing the predicted and true labels of lifecycle stage prediction when scPoli was trained on various subsets of the atlas and used to predict the remaining cells.

Fig. S6. Expression of top 5 bulk RNA-seq markers on the scRNA-seq atlas data

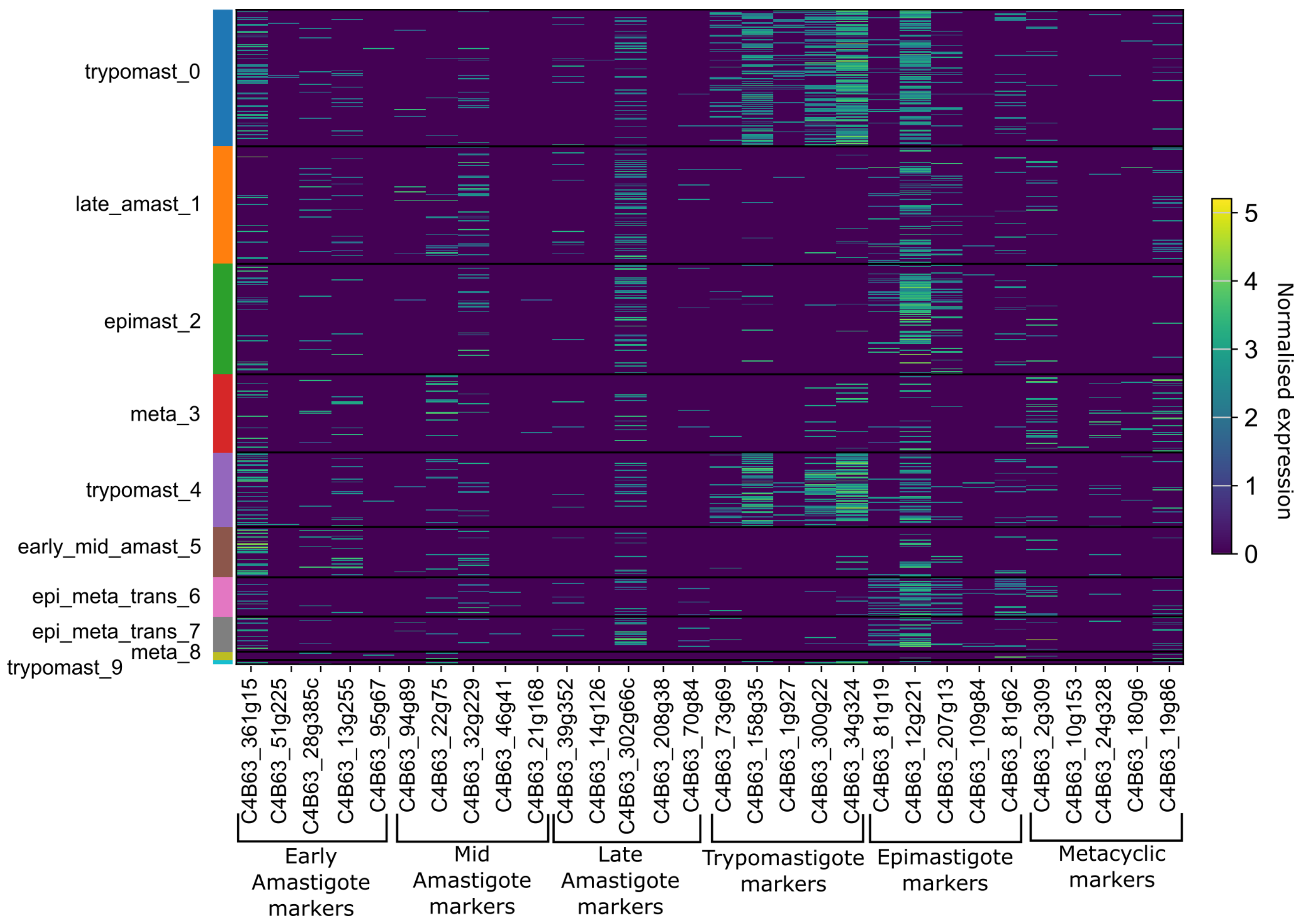

Heatmap showing the normalised expression of the top 5 bulk RNA-seq markers (as seen in fig. 2b) in the scRNA-seq atlas data

Fig. S7 Intersection of scRNA-seq cluster marker genes with bulk RNA-seq marker genes

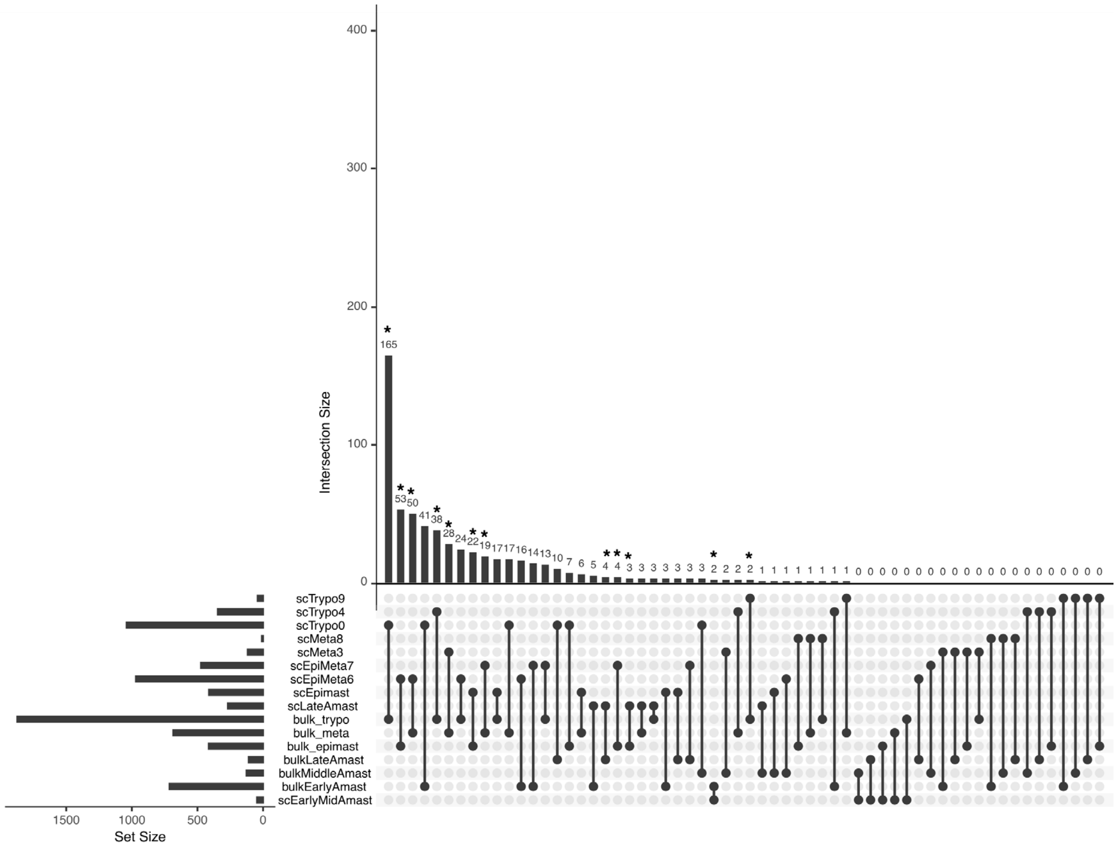

UpsetPlot showing the intersection of the marker genes of the scRNA-seq *T. cruzi* atlas clusters with the highly specific bulk RNA-seq marker genes with significant overlap of gene lists identified using a hypergeometric test with p-values adjusted for multiple testing using Bonferroni correction. * = Bonferroni corrected p-value < 0.05

Fig. S8. Single cell expression patterns of the 709 trans-sialidase genes.

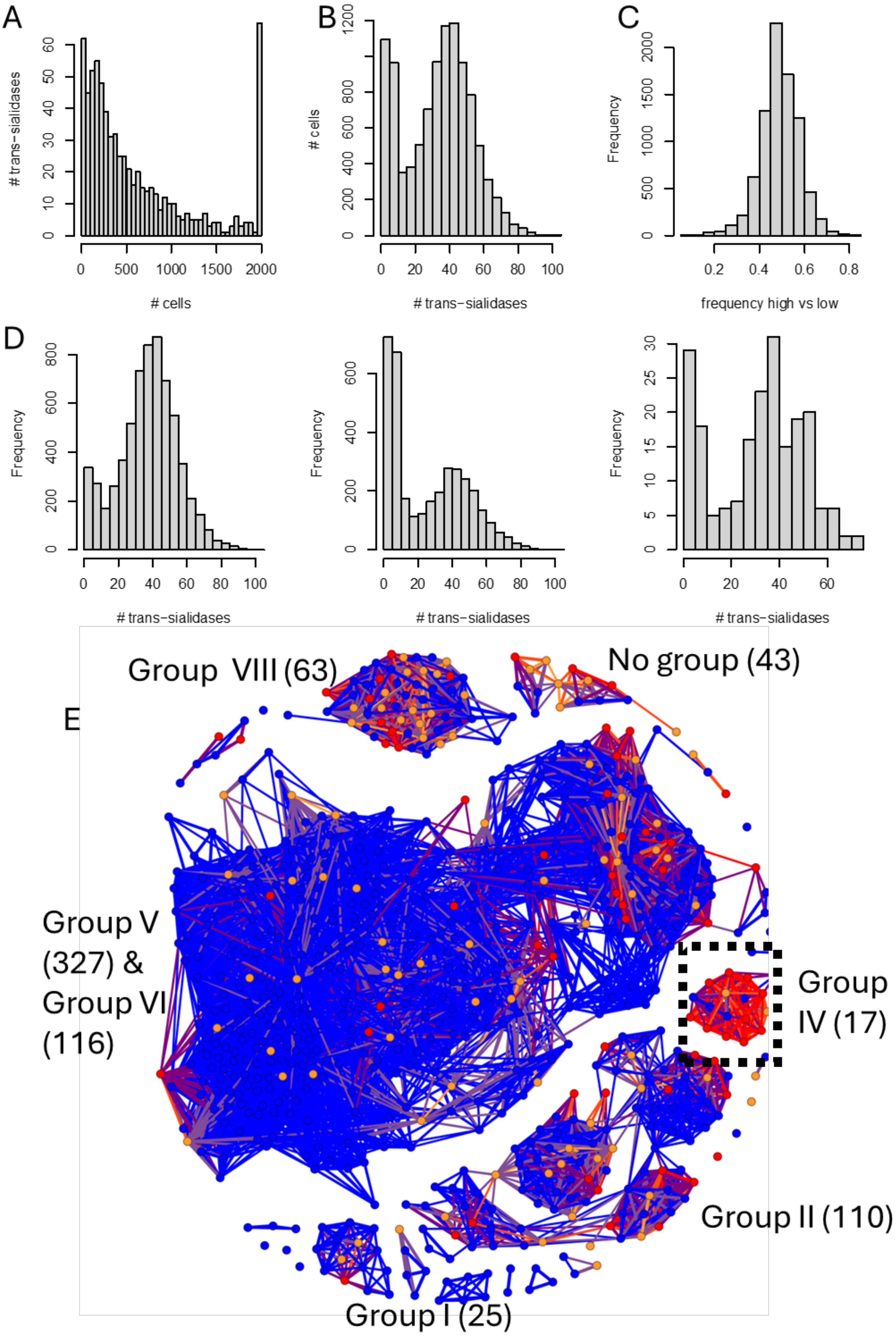

(A) Histogram of number of TS genes (Y-axis) expressed against number of cells expressing these (x-axis). Cut off at 2000 (65 TS). (B) Number of TS expressed per trypomastigote. (C) Frequency of high-expressed genes relative to total (of TS expressed in >20 cells). (D) same as B, but split for the three trypomastigote clusters; clusters 0, 4 and 9, from left to right. (E) Gephi network. Each dot represents a TS, colored by occurrence, in (A), red >2000, orange 1000-2000, blue < 1000 cells. TS are connected by global sequence similarity score > 61%. As indicated in the text, Group IV TS (black dotted box) TS occur in > 99% trypomastigotes (with at least 20 TS expressed). Five members that have > 85% identity to each other are C4B63_34g140, C4B63_109g60, C4B63_109g65, C4B63_34g137, C4B63_98g20. TS groups with more than 15 members are labelled (labels missing for Group III – 7 members & Group VII 2).

Fig. S9. Single cell expression patterns of the 736 mucin-associated surface proteins (MASP)

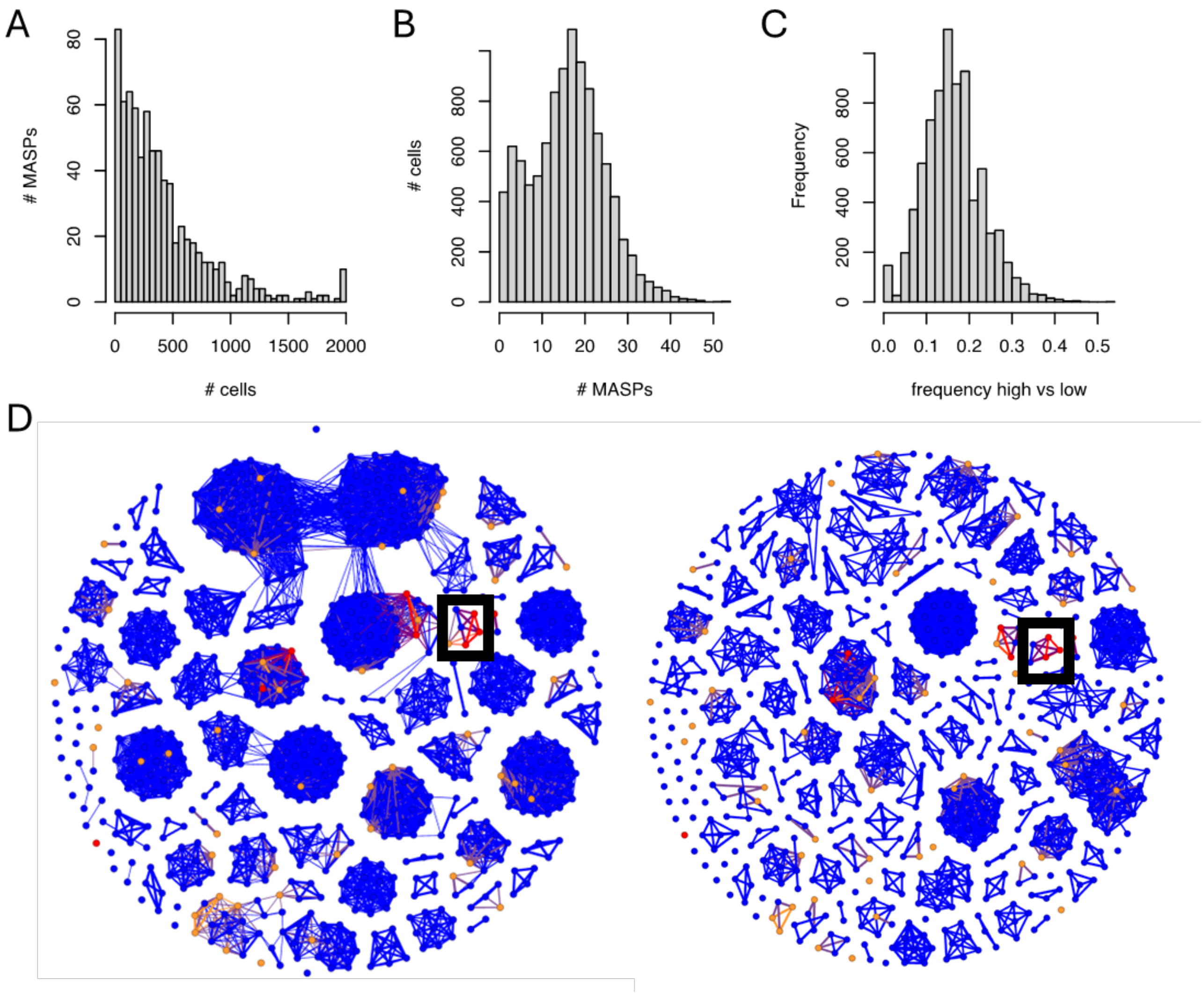

(A) Histogram of number of MASP genes (Y-axis) expressed against number of cells expressing these (x-axis). Cut off at 2000 (9 MASPs). (B) Number of MASPs expressed per trypomastigote. (C) Frequency of high-expressed genes relative to total (of MASPs expressed in >20 cells). (D) Gephi as in Supplementary Figure 3. Left identity cuf-off: >23%, right: >61%. Overall, the MASP gene families seem to be more diverse than the TS. Black box highlights three genes (C4B63_147g63, C4B63_196g38 and C4B63_24g26) and occur in 9773, 8865 and 4984 cells, respectively.

Fig. S10. Single cell expression patterns of other multi-gene family surface genes.

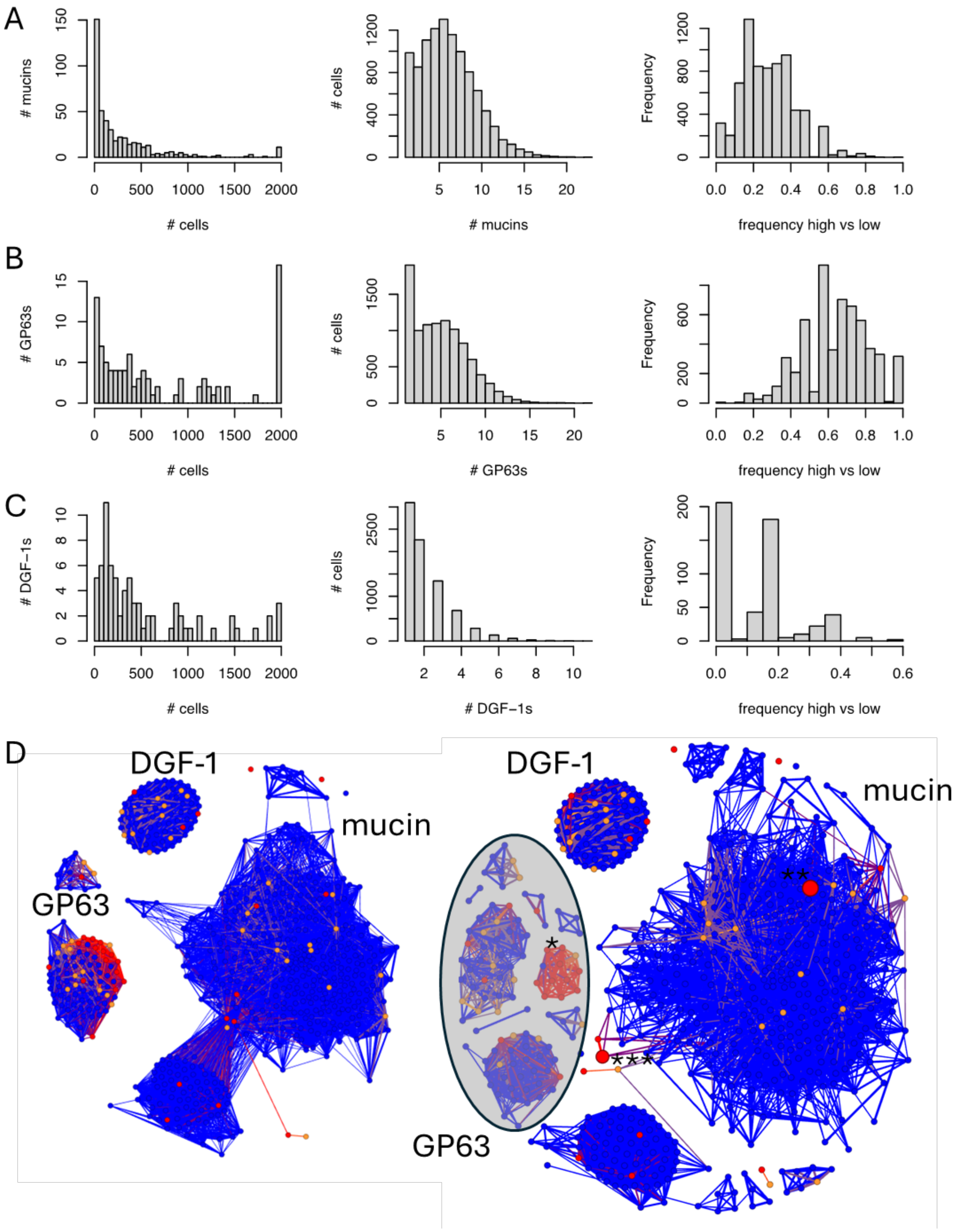

Expression pattern of 458 mucins (A), 96 Surface protease GP63 (B) and 75 dispersed gene family protein DGF-1 (C), see Supplementary Figure 3 for description. (D) Gephi graph for the three gene families with an identity cutoff of >21% (left) and >41% (right), as described in Supplementary Figure 3. At 21% identity, the three families cluster well together. At 41% the GP63 fall into several classes, with members expressed in more cells clustered together(*), as described for the other gene families before. However, for the mucins, C4B63_1g346 (**, large red dot) is expressed in 19,435 cells. C4B63_51g234 (second large dot ***), is expressed in 9,451 cells. They do not form a separate cluster of "conserved/highly expression" genes, compared to the other gene families. The DGF-1 genes are more conserved and share >80% sequence similarity.

Fig. S11. Visualisation of transcriptomics data in Artemis

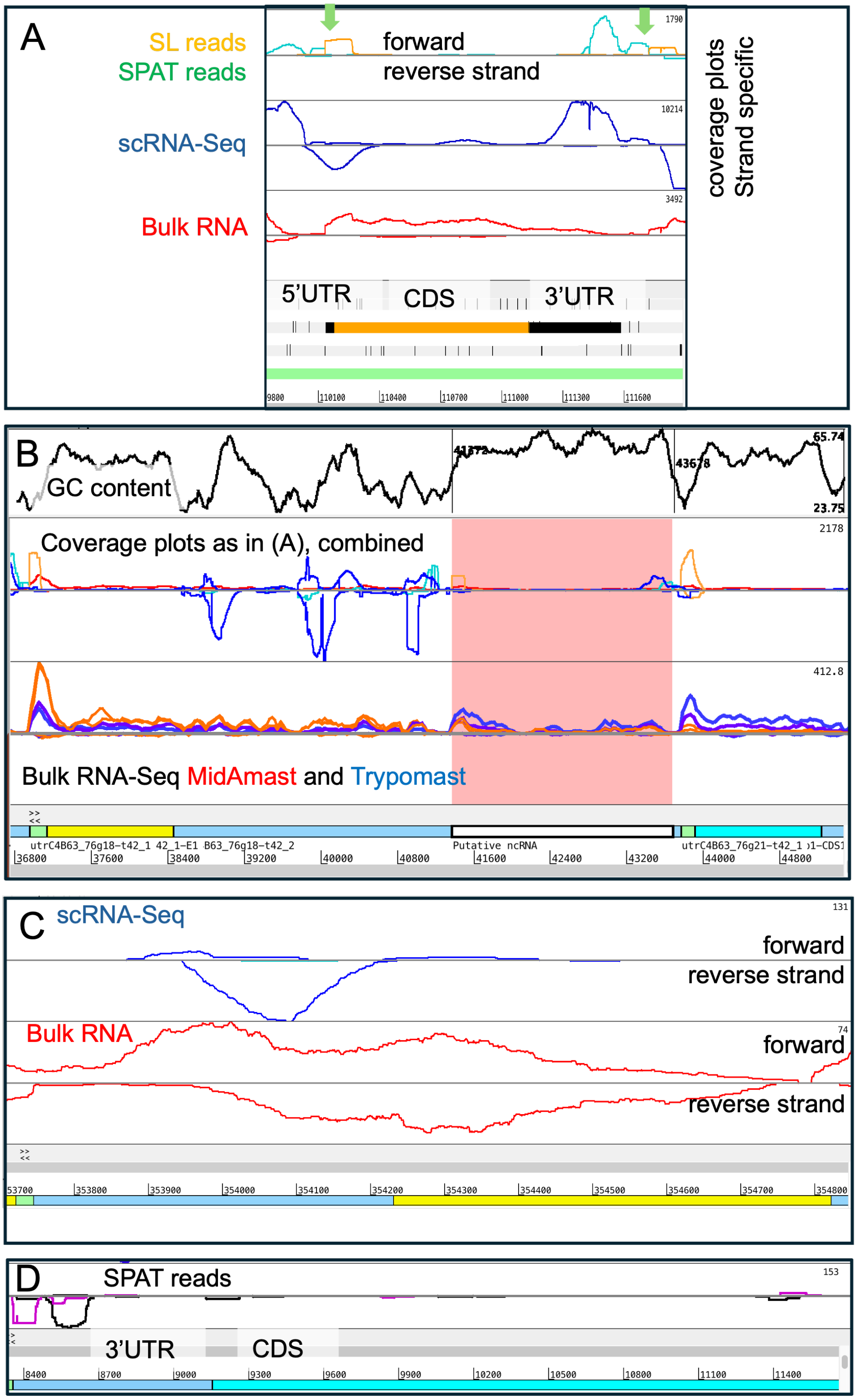

(A) In this long 5' UTR of C4B63_1g48, there is evidence of SL and SPAT (green arrows). The resulting open reading frame (ORF) of nearly 1000 bp has a perfect protein match (100% identity) against BCY84_06787 [*Trypanosoma cruzi* Dm28c 2017]. The red bulk RNA-Seq coverage plot shows evidence of expression, as well as the blue scRNA-Seq coverage plot. (B) Potential new ncRNA. On chromosome 76, in the 3'UTR of

C4B63_76g18, we find evidence for a novel transcript. In the bulk RNA-Seq we see evidence for different expression levels between MidAmast (bAMA35, red) and Trypomast (bADT, blue). It was not possible to build a gene model, and although this could be a pseudo gene, due to the high GC content and the lack of clear ORF, we speculate that this is a ncRNA. (C) Example of reverse strand expression of mucin (C4B63_11g381) genes. The gene is on the reverse strand, however on the forward strand, there is clear evidence of "reverse" transcription. Those could be ncRNA that are a further layer of transcriptional control, yet to be explored. Of the top 500 genes with evidence of transcription of the reverse strand (just using the exon, not UTR), 120 are of unknow function, 74 are MASPs, 26 DGF-1 and 24 mucins TcMUCII. (D) Example of alternative 3'UTR end in metacyclic trypomastigotes (violet) and amastigotes (black) in the gene C4B63_1g1402, a retrotransposon hot spot (RHS) protein. The UTR of is ~170bp longer in the metacyclic trypomastigote sample.

All coverage plots were filtered for a mapping score of at least 5 to exclude repetitive mapping reads.

Fig. S12. Extended UTR mapping (new mapping) vs 2500bp extended UTR mapping (old mapping).

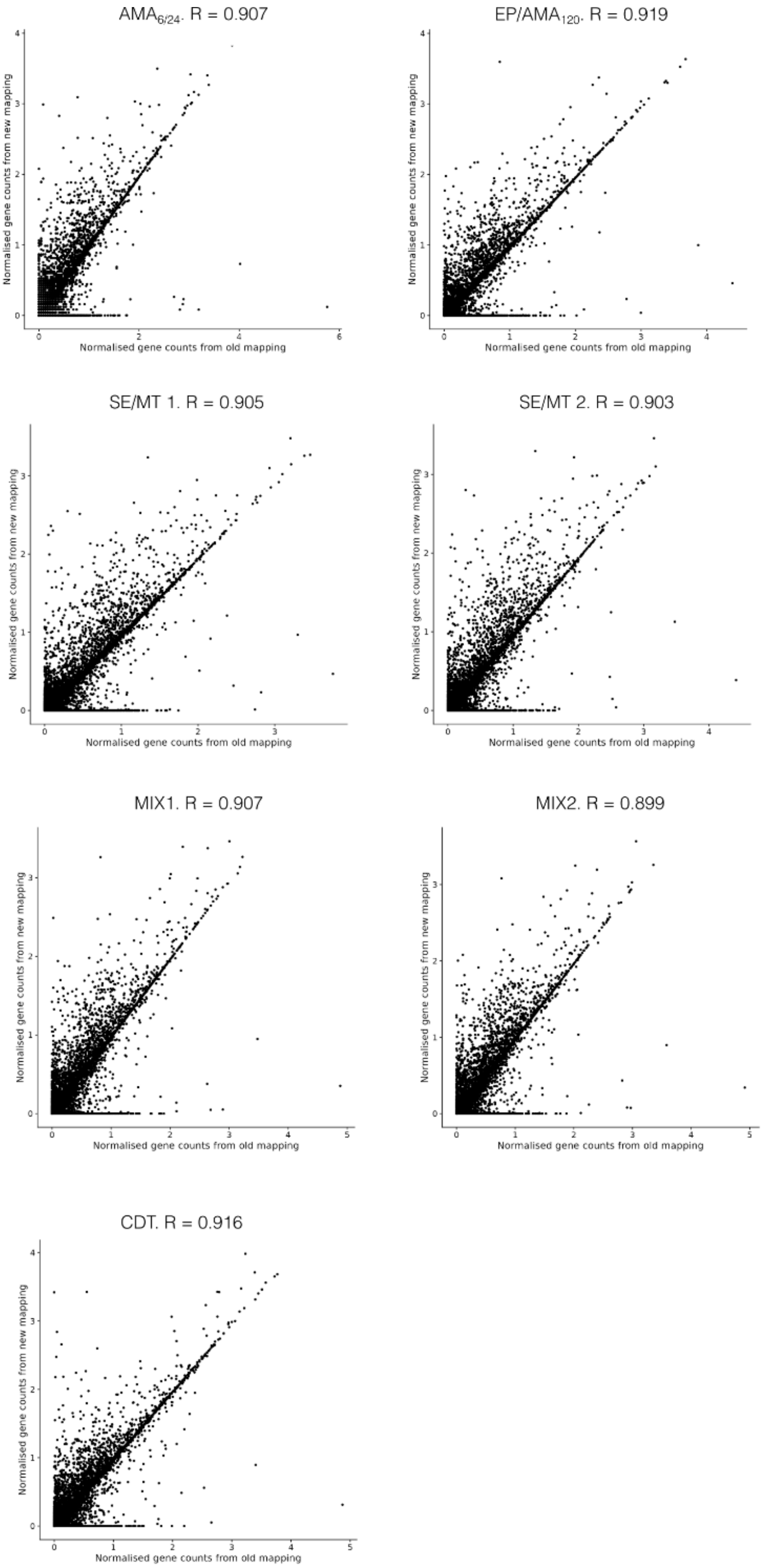

Relationship in normalised gene counts between the data extended UTR mapping (new mapping) and 2500bp extended UTR mapping (old mapping). The 10X sample and Pearson correlation coefficients are shown as the title of each plot.

Fig. S13. rRNA mapping

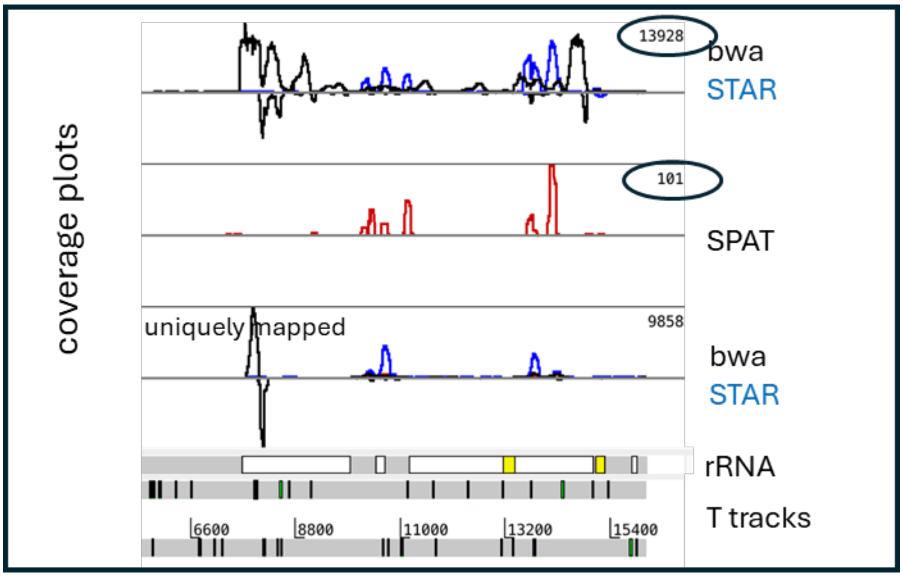

The white/yellow boxes are rRNA. The two plots how that the STAR mapper (blue lines), as part of cellRanger, maps far less reads compared to bwa (black line). There is no evidence of poly-adenylation of rRNA, as there are very few SPAT reads (<1%, noise). Rather, we hypothesize that due to the high abundance of rRNA in the parasite, small poly T/A tracks >5 bp (vertical lines, bottom of figure) are sufficient to anneal the rRNA to the capture oligos in the 10X reaction.

Table S1. Sample information for scRNA-seq samples

Sample Id	Stage collected	Viability (%)	No. cells/stage	No. cells/sample[c]	cDNA (ng/µl)[d]	library (ng/µl)[e]	Sequencing depth (Gb)
AMA6/24	AMA6h	91.6	8250	16500	2.2	8.1	220 Gb
	AMA24h	87.3	8250				
EP/AMA120	EP	98.9	8250	16500	2.7	14.5	220 Gb
	AMA120h	99.6	8250				
CDT	CDT	99.5	16500	16500	2.9	17.3	220 Gb
SE/MT[a]	SE/MT	97.8	16500	16500	0.8	7.8	70 Gb
		99.5	16500	16500	0.7	8.5	70 Gb
MIX[b]	AMA6h	96.4	3500	21000	0.8	24.8	200 Gb
	AMA24h	97.4	3500				
	AMA120h	98.9	3500				
	EP	98.2	3500	21000	0.7	29.2	200 Gb
	ADT	98.9	3500				
	SE/MT	96.7	3500				

[a] Two biological replicates collected

[b] Two technical replicates collected

[c] Number of cells loaded into the 10X Chromium Next Gem Chip G

[d] Diluted in 40 µl total volume

[e] Libraries prepared with 10 µl cDNA; diluted in 35 µl total volume

Table S2. Comparison of mapping stats for Dm28c 2018 and Sylvio X10 2017 for the MIX 1 dataset

Reference genome[a]	Median genes per cell	No. cells	% reads mapping to the genome	% reads mapping confidently to transcriptome
Dm28c 2018	216	5464	55.0	4.7
Sylvio X10 2017	135	5804	15.8	2.0

[a] The genomes have not been altered in any way from when they were downloaded from TriTrypDB (i.e. no UTR extension).

Table S3. Selected statistics generated by 10X Cellranger output for scRNA-seq samples

Sample Id	No. *T. cruzi* cells recovered	Total reads	Total reads *T. cruzi*[a]	Median genes *T. cruzi*	% reads mapping confidently to:				
					T. cruzi transc (raw)	*T. cruzi* transc (adjusted)	human transc	*T. cruzi* genome	human genome
AMA6/24	1162	952950562	387850879	307	1.6	3.9	33.1	6.3	59.3
EP/AMA120	6434	682978286	678197438	928	14.4	14.5	0.2	43.1	0.7
CDT	7978	729447233	729447233	762	7.7	7.7	NA	30.1	NA
SE/MT 1	4424	271060069	271060069	298	18.4	18.4	NA	44.4	NA
SE/MT 2	3582	251411209	251411209	421	21.4	21.4	NA	57.4	NA
MIX 1	5393	674582011	611171302	503	10.1	11.2	6.6	56.2	9.4
MIX 2	5609	739240858	663838290	422	9.9	11.0	7.3	56.8	10.2

[a] For datasets which contain human cells, 'total reads *T. cruzi*' was calculated by assuming all human reads will map to the human genome and the rest of the reads will be from the *T. cruzi* samples. This was then used to calculate the '% of reads mapping confidently to *T. cruzi* transc adjusted' values. Transc: transcriptome.

Table S4. Cutoffs used for filtering out low quality cells across the scRNA-seq samples

Sample Id	Gene counts minimum	Gene counts maximum	% reads mapping to *T. cruzi* *mitochondrial* genes maximum	% reads mapping to human transcriptome maximum
AMA6/24	200	750	20	50
EP/AMA120	400	2000	30	10
CDT	200	1500	25	NA
SE/MT 1	150	1000	40	NA
SE/MT 2	200	1250	40	NA
MIX 1	150	1500	28	30
MIX 2	200	1250	30	30

Table S5. F1-scores for the scPoli predictions across the 10 atlas clusters, for the different subsets

Cluster Id	Model trained on 95% of atlas R eplicate 1	Model trained on 95% of atlas Replicate 2	Model trained on 75% of atlas Replicate 1	Model trained on 75% of atlas Replicate 2	Model trained on 50% of atlas Replicate 1	Model trained on 50% of atlas Replicate 2	Model trained on 25% of atlas Replicate 1	Model trained on 25% of atlas Replicate 2
early_mid_amast_5	0.80	0.74	0.73	0.73	0.71	0.73	0.63	0.62
epi_meta_trans_6	0.75	0.77	0.76	0.75	0.75	0.76	0.70	0.65
epi_meta_trans_7	0.59	0.61	0.59	0.70	0.60	0.63	0.54	0.57
epimast_2	0.87	0.85	0.86	0.85	0.83	0.84	0.82	0.81
late_amast_1	0.80	0.77	0.76	0.79	0.73	0.74	0.69	0.66
meta_3	0.85	0.82	0.79	0.78	0.79	0.78	0.76	0.74
meta_8	0.79	0.75	0.79	0.79	0.74	0.66	0.63	0.66
trypomast_0	0.76	0.78	0.75	0.75	0.75	0.75	0.73	0.73
trypomast_4	0.72	0.75	0.72	0.73	0.70	0.70	0.68	0.68
trypomast_9	0.64	0.70	0.68	0.69	0.73	0.41	0.63	0.60

Table S6. Sample information for bulk RNA-seq samples

Sample Id	No. parasites collected[a]	RNA (ng/µl[b])	RNA (ng/1E+06 parasites)	mean RNA/stage (ng/1E+06 parasites)
bAMA6	8.40E+06	56	200	185.8
	7.52E+06	43	171.5	
bAMA24	3.76E+06	22	175.5	146.9
	3.81E+06	15	118.3	
bAMA120	1.16E+07	70	181	163.2
	1.94E+07	94	145.4	
bEP	4.00E+07	265	198.8	213.8
	4.00E+07	305	228.8	
bCDT	4.00E+07	89	66.8	65.8
	4.00E+07	72	54	
	4.00E+07	102	76.5	
bSE/MT	4.00E+07	59	44.3	39
	4.00E+07	45	33.8	

[a] Two biological replicates collected per sample, with two technical replicates prepared for the second bADT biological replicate

[b] Diluted in 30 µl total volume

Table S7. Mapping statistics of the bulk RNA sequencing samples mapped against the Dm28c *T. cruzi* + kDNA genome

Sample Id	No. input reads[a]	% uniquely mapped reads	% reads mapped to multiple loci	% reads mapped to too many loci	% reads unmapped: too many mismatches	% reads unmapped: too short	% reads unmapped: other	Total alignments (featureCounts)	% successfully assigned alignments (featureCounts)
bAMA6	23475929	41.88	14.48	9.83	0	31.79	2.02	21942505	38.20
	20219803	46.33	16.26	10.92	0	24.33	2.17	21107762	38.20
bAMA24	20498527	36.14	12.46	5.11	0	44.57	1.73	16395372	38.10
	24492333	35.19	12.00	4.52	0	46.42	1.87	18848721	39.50
bAMA120	26077005	52.40	17.93	16.22	0	9.89	3.56	30285994	36.40
	27749110	49.86	17.13	21.31	0	8.70	2.99	31157951	35.00
bEP	25717422	54.56	19.39	18.14	0	4.01	3.90	32736362	34.30
	25985438	56.38	18.93	16.58	0	3.91	4.20	32444213	36.80
bCDT	27090531	51.03	20.71	22.63	0	3.91	1.73	34960443	31.40
	21882358	48.82	20.93	22.23	0	5.18	2.84	27812588	31.80
bEP 2	22048702	43.12	19.93	28.85	0	5.46	2.64	26768688	29.30
bSE/MT	22249655	59.79	18.83	14.13	0	4.02	3.23	28940501	36.50
	28225858	64.08	18.66	9.96	0	4.78	2.52	36415986	39.50

[a] Two biological replicates were collected per sample, with two technical replicates prepared for the second bADT biological replicate

Supplementary Table 8. Selected statistics generated by 10X Cellranger output for scRNA-seq samples, mapped to the new UTR extended reference

Sample Id	No. *T. cruzi* cells recovered	Total reads	Total reads *T. cruzi*[a]	Median genes *T. cruzi*	% reads mapping confidently to:				
					T. cruzi transc (raw)	*T. cruzi* transc (adjusted)	human transc	*T. cruzi* genome	human genome
AMA6/24	1175	952950562	394521533	296	1.2	2.89	31.2	2.8	46.6
EP/AMA120	6397	682978286	680246373	812	11.1	11.14	0.2	22.5	0.4
CDT	7915	729447233	729447233	670	6.9	6.9	NA	15.3	NA
SE/MT 1	4632	271060069	271060069	267	13.0	13.0	NA	28.1	NA
SE/MT 2	3592	251411209	251411209	408	13.4	13.4	NA	32.0	NA
MIX 1	5506	674582011	617917122	473	7.9	8.62	6.0	15.1	8.4
MIX 2	5591	739240858	670491458	411	7.9	8.71	6.7	14.6	9.3

[a] For datasets which contain human cells, 'total reads *T. cruzi*' was calculated by assuming all human reads will map to the human genome and the rest of the reads will be from the *T. cruzi* samples. This was then used to calculate the '% of reads mapping confidently to *T. cruzi* transc adjusted' values. Transc: transcriptome.
