# Supplementary material for: Trypanosoma cruzi cell atlas as a single-cell resource for understanding parasite population heterogeneity and differentiation
Source: Nat Commun. 2026 May 25;17:6801. doi: 10.1038/s41467-026-73098-w (PMC13385397; doi:10.1038/s41467-026-73098-w)
Supplement: Supplementary file 3 — Description of Additional Supplementary Files [file 41467_2026_73098_MOESM3_ESM.pdf]

**Description of Additional Supplementary Files**

File Name: Supplementary Data 1
Description: Marker genes for scRNA-seq clusters
scores: Z-score from p-value
logFC: log fold change between the clusters
pvals: Mann-Whitney U test p-value
pvals_adj: Bonferroni adjusted p-value

File Name: Supplementary Data 2
Description: Parasite stage-specific marker genes identified by bulk RNA-seq
baseMean: Average of normalized counts
log2FC: Log2 fold change of gene expression for the defined contrast
lfcSE: Standard error estimate for the log2FC measurement
stat: Wald statistic
pvalue: Wald test p-value
padj: Bonferroni adjusted p-value

File Name: Supplementary Data 3
Description: Amastigote stage-specific marker genes identified by bulk RNA-seq
baseMean: Average of normalized counts
log2FC: Log2 fold change of gene expression for the defined contrast
lfcSE: Standard error estimate for the log2FC measurement
stat: Wald statistic
pvalue: Wald test p-value
padj: Bonferroni adjusted p-value

File Name: Supplementary Data 4
Description: Marker genes for trypomastigote subpopulations identified by single-cell RNA-seq
scores: Z-score from p-value
logFC: log fold change between the clusters
pvals: Mann-Whitney U test p-value
pvals_adj: Bonferroni adjusted p-value

File Name: Supplementary Data 5
Description: Genes associated with the epimastigote to metacyclic trypomastigote transition
RBP?: Does the gene encode an RNA-binding protein?
Cluster phase: The point in the phase where the gene peaks in expression, according to the tradeSeq model
meanLogFC: Average log fold change across the knots of the tradeSeq model
pvalue: Wald test p-value
p_adjust: Bonferroni adjusted p-value/p
