# Supplementary material for: Trypanosoma cruzi cell atlas as a single-cell resource for understanding parasite population heterogeneity and differentiation
Source: Nat Commun. 2026 May 25;17:6801. doi: 10.1038/s41467-026-73098-w (PMC13385397; doi:10.1038/s41467-026-73098-w)
Supplement: Supplementary file 9 — Reporting Summary [file 41467_2026_73098_MOESM9_ESM.pdf]

nature portfolio

Corresponding author(s): Thomas Otto and Manu De Rycker

Last updated by author(s): 14/1/2025

Reporting Summary

Nature Portfolio wishes to improve the reproducibility of the work that we publish. This form provides structure for consistency and transparency in reporting. For further information on Nature Portfolio policies, see our Editorial Policies and the Editorial Policy Checklist.

Statistics

For all statistical analyses, confirm that the following items are present in the figure legend, table legend, main text, or Methods section.

n/a	Confirmed	
☐	☒	The exact sample size (*n*) for each experimental group/condition, given as a discrete number and unit of measurement
☐	☒	A statement on whether measurements were taken from distinct samples or whether the same sample was measured repeatedly
☐	☒	The statistical test(s) used AND whether they are one- or two-sided<br>*Only common tests should be described solely by name; describe more complex techniques in the Methods section.*
☒	☐	A description of all covariates tested
☐	☒	A description of any assumptions or corrections, such as tests of normality and adjustment for multiple comparisons
☒	☐	A full description of the statistical parameters including central tendency (e.g. means) or other basic estimates (e.g. regression coefficient) AND variation (e.g. standard deviation) or associated estimates of uncertainty (e.g. confidence intervals)
☒	☐	For null hypothesis testing, the test statistic (e.g. *F*, *t*, *r*) with confidence intervals, effect sizes, degrees of freedom and *P* value noted<br>*Give P values as exact values whenever suitable.*
☒	☐	For Bayesian analysis, information on the choice of priors and Markov chain Monte Carlo settings
☒	☐	For hierarchical and complex designs, identification of the appropriate level for tests and full reporting of outcomes
☒	☐	Estimates of effect sizes (e.g. Cohen's *d*, Pearson's *r*), indicating how they were calculated

Our web collection on statistics for biologists contains articles on many of the points above.

Software and code

Policy information about availability of computer code

Data collection: CytExpert version 2.4

Data analysis: 10x Cell Ranger v6.0.0.

STAR version 2.7.11a
FeatureCounts version 2.0.6; Peaks2UTR 1.4.0; BWA 0.7.17-r1188
Analyses in Python were carried out on Python version 3.8.16. Specific package versions for Python analysis are as follows: anndata – 0.9.1, h5py – 3.8.0, leidenalg – 0.10.1, matplotlib – 3.7.1, numpy – 1.24.3, pandas – 2.0.3, pip – 23.0.1, scanpy – 1.9.3, scvi-tools – 0.20.3, scArches – 0.5.10, seaborn – 0.12.2, scipy – 1.19.1, scikit-learn – 1.2.2, umap-learn – 0.5.3, bbknn – 1.6.0, fa2 – 0.3.5, igraph – 0.10.6, pytorch-lightning - 1.9.5

Analyses in R were carried out on R version 4.1.0. Specific package versions for R analysis are as follows: singlecellexperiment – 1.14.1, tradeSeq – 1.6.0, metR – 0.15.0, ComplexHeatmap – 2.8.0

For manuscripts utilizing custom algorithms or software that are central to the research but not yet described in published literature, software must be made available to editors and reviewers. We strongly encourage code deposition in a community repository (e.g. GitHub). See the Nature Portfolio guidelines for submitting code & software for further information.

Data

Policy information about availability of data

All manuscripts must include a data availability statement. This statement should provide the following information, where applicable:

- Accession codes, unique identifiers, or web links for publicly available datasets
- A description of any restrictions on data availability
- For clinical datasets or third party data, please ensure that the statement adheres to our policy

Raw sequencing data is available through ArrayExpress, accession numbers E-MTAB-14400 (bulk) and E-MTAB-14406 (single-cell). All UTR annotation data can be found on Zenodo (https://zenodo.org/records/14229160). scRNA-Seq data can be queried here: https://cellatlas-cxg.mvls.gla.ac.uk/Trypanosoma_cruzi_inVitro_cellatlas/.

Research involving human participants, their data, or biological material

Policy information about studies with human participants or human data. See also policy information about sex, gender (identity/presentation), and sexual orientation and race, ethnicity and racism.

Reporting on sex and gender	No research involving human participants.
Reporting on race, ethnicity, or other socially relevant groupings	No research involving human participants.
Population characteristics	No research involving human participants.
Recruitment	No research involving human participants.
Ethics oversight	No research involving human participants.

Note that full information on the approval of the study protocol must also be provided in the manuscript.

Field-specific reporting

Please select the one below that is the best fit for your research. If you are not sure, read the appropriate sections before making your selection.

☒ Life sciences ☐ Behavioural & social sciences ☐ Ecological, evolutionary & environmental sciences

For a reference copy of the document with all sections, see nature.com/documents/nr-reporting-summary-flat.pdf

Life sciences study design

All studies must disclose on these points even when the disclosure is negative.

Sample size	No statistical method was used to determine sample size. For single cell experiments, at least 16,500 cells were used per condition, this is higher than the 10x Genomics recommendation of a maximum of 10,000 cells, and is based on our and other's findings that for small protozoan cells, better results are obtained with larger cell numbers. For bulk RNA-seq experiments 1 µg total RNA was used per sample for library preparation, as per recommendation by the sequencing provider.
Data exclusions	No data was excluded from the analyses.
Replication	We applied a replication strategy that balances robustness with cost of experiments. The scRNA-seq replication strategy is detailed in Figure 1 and included generating samples of individual life cycle stages, or small pools, as well as a replicate that included a mixture of all tested life cycle stages.
Randomization	Randomization was not applicable to this study, as specific life cycle stages were analyzed.
Blinding	Blinding was not possible, as the different life cycle stages of the parasite show distinct and easily observable morphologies.

Reporting for specific materials, systems and methods

We require information from authors about some types of materials, experimental systems and methods used in many studies. Here, indicate whether each material, system or method listed is relevant to your study. If you are not sure if a list item applies to your research, read the appropriate section before selecting a response.

Materials & experimental systems

n/a	Involved in the study
☒	☐ Antibodies
☐	☒ Eukaryotic cell lines
☒	☐ Palaeontology and archaeology
☒	☐ Animals and other organisms
☒	☐ Clinical data
☒	☐ Dual use research of concern
☒	☐ Plants

Methods

n/a	Involved in the study
☒	☐ ChIP-seq
☐	☒ Flow cytometry
☒	☐ MRI-based neuroimaging

Eukaryotic cell lines

Policy information about cell lines and Sex and Gender in Research

Cell line source(s)	Vero cells were obtained from the European Collection of Authenticated Cell Cultures (catalog 84113001). No further authentification was carried out. The Vero cell line was established from the kidney of an African green monkey in 1962. Recent genomic analysis indicated that the line is female, and species is Chlorocebus sabaeus.<br>T. cruzi Silvio parasites were used (MHOM/BR/78/Silvio; clone X10/7-A1). The Silvio strain was originally isolated from a 19-year-old male patient (Silvio B.S.) living in Pará, Brazil (Silveira F. T., Viana Dias M. G., Pereira Pardal P., Oliveira Lobâo A., Britto Melo G. Nono caso-autóctone de doença de Chagas registrado no estado do Pará, Brasil (Nota prévia) Hiléia Médica. 1979;1:61–62.).
Authentication	No authentication was carried out for the Vero cells. For T. cruzi X10/7 cells we authenticated the correct Discrete Typing Unit using Single Nucleotide Polymorphism genotyping.
Mycoplasma contamination	Vero cells were certified mycoplasma free as provided by the European Collection of Authenticated Cell Cultures. T. cruzi parasites were not mycoplasma tested.
Commonly misidentified lines (See ICLAC register)	*Name any commonly misidentified cell lines used in the study and provide a rationale for their use.*

Plants

Seed stocks	*Report on the source of all seed stocks or other plant material used. If applicable, state the seed stock centre and catalogue number. If plant specimens were collected from the field, describe the collection location, date and sampling procedures.*
Novel plant genotypes	*Describe the methods by which all novel plant genotypes were produced. This includes those generated by transgenic approaches, gene editing, chemical/radiation-based mutagenesis and hybridization. For transgenic lines, describe the transformation method, the number of independent lines analyzed and the generation upon which experiments were performed. For gene-edited lines, describe the editor used, the endogenous sequence targeted for editing, the targeting guide RNA sequence (if applicable) and how the editor was applied.*
Authentication	*Describe any authentication procedures for each seed stock used or novel genotype generated. Describe any experiments used to assess the effect of a mutation and, where applicable, how potential secondary effects (e.g. second site T-DNA insertions, mosiacism, off-target gene editing) were examined.*

Flow Cytometry

Plots

Confirm that:

- ☒ The axis labels state the marker and fluorochrome used (e.g. CD4-FITC).
- ☒ The axis scales are clearly visible. Include numbers along axes only for bottom left plot of group (a 'group' is an analysis of identical markers).
- ☐ All plots are contour plots with outliers or pseudocolor plots.
- ☒ A numerical value for number of cells or percentage (with statistics) is provided.

Methodology

Sample preparation	Aliquots from all parasite stages diluted at a density of 1x106 cells/ml were stained for 5 min at room temperature with SYTOX™ AADvanced™ Dead Cell Stain (Life Technologies, Eugene, OR, USA) following manufacturers' recommendations. A total of 20,000 events per sample were acquired with excitation at 488 nm and emission at 647 nm wavelengths. Parasites killed with three cycles of freezing and thawing were used as positive control for SYTOX AADvanced staining.
Instrument	CytoFLEX S (Beckman Coulter, Indianapolis, IN, USA)

Software	CytExpert version 2.4
Cell population abundance	No sorting carried out.
Gating strategy	All gates (including FSC/SSC) are shown on the figure. Threshold for identifying dead cells was determined using dead parasites.

☒ Tick this box to confirm that a figure exemplifying the gating strategy is provided in the Supplementary Information.
