# Supplementary material for: Trypanosoma cruzi cell atlas as a single-cell resource for understanding parasite population heterogeneity and differentiation
Source: Nat Commun. 2026 May 25;17:6801. doi: 10.1038/s41467-026-73098-w (PMC13385397; doi:10.1038/s41467-026-73098-w)
Supplement: Supplementary file 2 — Peer Review file [file 41467_2026_73098_MOESM2_ESM.pdf]

nature portfolio

Peer Review File

Trypanosoma cruzi cell atlas as a single-cell resource for understanding parasite population heterogeneity and differentiation

Corresponding Author: Dr Manu De Rycker

This file contains all reviewer reports in order by version, followed by all author rebuttals in order by version.

Attachments originally included by the reviewers as part of their assessment can be found at the end of this file.

Version 0:

Reviewer comments:

Reviewer #1

(Remarks to the Author)
This manuscript presents a comprehensive scRNA-seqatlas of the Trypanosoma cruzi life cycle, complemented by bulk RNA-seq data. The study offers detailed insights into gene expression dynamics across different life cycle stages and transitions, with a particular focus on the differentiation from epimastigote to metacyclic trypomastigote. The authors provide extensive UTR annotation and analyze multi-gene family expression at the single-cell level. Through trajectory analysis, they identify more than 900 genes associated with the epimastigote-to-metacyclic trypomastigote transition, including about 30 RNA-binding proteins. Strikingly, two distinct trypomastigote subpopulations emerge, one characterized by higher expression of metabolism-related genes and the other by elevated expression of surface protein genes (the latter being really surprising). Furthermore, single-cell analysis of multi-gene family expression reveals cell-specific repertoires of surface antigens in trypomastigotes.
This study represents a wonderful advance in our understanding of T. cruzi biology and is highly suitable for publication in Nature Communications. Below are some points for potential consideration, which I am confident the authors have already thought of:
1. The transition from metacyclic trypomastigote to amastigote warrants more discussion.
2. The implications of the observed alternative polyadenylation and antisense transcription patterns for gene regulation could be elaborated in greater depth.
3. Investigating the functional significance of the two trypomastigote subpopulations would be highly valuable.
4. The potential regulatory roles of alternative polyadenylation sites and antisense transcripts merit experimental follow-up.
While addressing all these points would require extensive additional experimentation, I do not believe such work should be requested as a prerequisite for publication.
In conclusion, this manuscript presents an important and carefully executed study that substantially enhances our understanding of T. cruzi gene expression dynamics across its life cycle. The data and analyses provided offer a rich resource for future research. While some aspects of gene regulation and life cycle transitions remain to be fully elucidated, the current findings are already highly compelling. Pending very minor revisions, this work is well-suited for publication in Nature Communications.

Reviewer #2

(Remarks to the Author)
The report is attached.

Reviewer #3

(Remarks to the Author)
The authors present a well-executed study using single-cell transcriptomics to analyze the different life stages of Trypanosoma cruzi. This is a first single-cell transcriptomic data set for the protozoan parasite T. cruzi and will certainly

provide a valuable resource for the research community. The manuscript presents a single-cell atlas for in-vitro grown parasites, allowing identification of clusters of epimastigotes, metacyclic trypomastigotes, transitionary stages between epimastigotes and metacyclic trypomastigotes, amastigote-derived trypomastigotes, and amastigotes at different stage of infection. It provides novel insights into the heterogeneity of different life stages by detecting transcriptionally different populations of trypomastigotes and describing different cell-specific multi-gene family repertoires. In addition, it presents a bulk RNAseq dataset with markers of the in vitro-derived life stages studied here. Finally, by combining scRNAseq and bulk RNAsed data it maps UTRs, it identifies alternative polyadenylation sites, and reverse strand and antisense transcripts.

General comments

One of the main deliverables of the manuscript is the generation of the complete T. cruzi cell atlas, while actually not all transitions between life stages, and potentially not all the life stages are being described. The authors focus their trajectory analysis solely on the transition between epimastigotes and metacyclics, and the transition from the metacyclic trypomastigote to amastigote is missing. However, if the single-cell RNA-seq dataset captures all life stages, a full trajectory analysis should, in principle, allow for the reconstruction of all developmental transitions. As such the cell atlas cannot be called “complete”. In addition, dormant amastigotes seem to be missing, even though their existence and their potential role for the development of novel therapeutics is mentioned by the authors in the introduction. It is not clear whether “late_amast” could be considered dormant or dormant amastigotes were not detected in the dataset?

Overall, the manuscript focuses on annotating the main life stages in the single-cell atlas, and confirming the correctness of the annotation, but it does not provide novel insights into biology of T. cruzi. There is no experimental validation of the findings: no novel life-stage specific markers are experimentally validated or no functional verification of newly described genes regulating the transition from epimastigotes to metacyclis has been done. Also, no evident novel biological hypothesis based on the single cell transcriptome findings are presented here.

Metacyclic trypomastigotes and trajectory analysis: in Fig 2a, two populations of metacyclic trypomastigotes (meta_8 and meta_3) are detected and the markers associated with these two clusters are presented in supplementary file 1. Then, later in the manuscript a trajectory analysis of the transition from epimastigotes to metacyclics is presented. It is not clear how these two populations of metacyclics are linked to the trajectory analysis. Would one population be early metacyclics and the other one late? Are they functionally different? Are they both non-replicative? Recent paper in Leishmania described two populations of metacyclics with early ones surprisingly being replicative, and the late ones not (Catta-Preta et al., 2024). Is a similar scenario possible here?

While the bioinformatics analyses presented are of high quality, some methodological decisions are not always clearly justified. Additionally, certain key details are missing from the supplementary tables.
• Doublet Detection: The authors use the number of genes expressed as an indicator of doublets. However, this is a rather rudimentary approach, given that more sophisticated methods, such as Scrublet, are available. This method also risks excluding highly transcriptionally active cells while failing to filter out some true doublets. Furthermore, considering the variability in the number of genes expressed across different samples, does applying a fixed threshold make sense, or should this criterion be adjusted per sample? Since some samples contain a mix of human and T. cruzi cells, a more precise estimate of doublet occurrence should be possible, allowing for a more data-driven threshold in doublet detection.
• Supplementary Table 1: While the number of cells loaded onto the 10X platform is reported, it would also be valuable to include the number of cells recovered for each dataset. Additionally, the number of UMIs per cell after filtering is not mentioned in any supplementary table.
• Supplementary Table 3: Different cut-offs were applied depending on the sample analyzed. While this approach is reasonable, it raises the concern that clustering may primarily reflect differences in transcriptional activity or read depth per cell rather than true gene-level transcriptional differences.
• Supplementary Table 4: The rationale for imposing a maximum threshold on the number of reads mapping to T. cruzi is unclear. In samples consisting solely of T. cruzi, one would expect the fraction of reads mapping to its genome to be as close to 100% as possible. Could the authors clarify this decision?

The 3' UTR prediction based on the transcriptome data presented in this study is a valuable effort. However, it is unclear why the authors used a relatively rough approach—defining the 3' UTR region by extending 2,500 bp beyond the gene's end position—for the scRNA-seq analysis when a high-quality list of 3' UTR regions is made available within their work. Could they clarify the reasoning behind this choice?

The added value of the bulk RNA-seq data in this study is not entirely clear. The authors appear to place significant emphasis on previously identified biomarkers rather than using the bulk RNA-seq results to derive novel insights. To better justify the integration of bulk RNA-seq with scRNA-seq, the authors should provide additional details regarding the overlap between scRNA-seq marker genes and differentially expressed genes from bulk RNA-seq. Specifically, they should assess whether this overlap is statistically significant (e.g., using a hypergeometric test). Furthermore, it would be informative to visualize the top expressed genes from bulk RNA-seq on the scRNA-seq UMAP projections to determine how well bulk-dominant genes map onto single-cell clusters. Additionally, the authors should explore the relationship between bulk RNA-seq expression levels and scRNA-seq marker recovery—do the scRNA-seq markers predominantly represent highly expressed genes from bulk RNA-seq, or does scRNA-seq also capture lowly expressed genes that might be biologically relevant?
Moreover, already 5 papers with bulk RNAseq of different T. cruzi life stages are already published (and are mentioned in the supplementary file 2), hence the novelty of and the need for the current bulk RNAseq datset is not evident.

scPoli predictions: While the authors clearly describe their approach to training and testing the life-stage classifier, the reported results are less convincing. The average F1 score occasionally drops to 0.60, and for certain life stages, it falls as low as 0.30—values that are considerably lower than the ≥0.80 F1-score typically expected in machine learning evaluations. Moreover, the classifier is validated on a sample generated using the same experimental setup as the training data. This raises concerns about its generalizability—how well it would perform in more distinct settings, such as different species or alternative single-cell platforms. To better assess performance, presenting the confusion matrix would be valuable. Additionally, do the authors have insights into the key features driving the classification? It remains unclear whether the classifier is leveraging biologically meaningful gene expression signatures or if it is primarily responding to overall transcriptional activity, which could reduce its ability to distinguish life stages robustly.

Validation of the cell atlas as a reference map for other datasets: it is not clear what kind of sample was used to validate the atlas. Was it generated using the same strain with exactly the same protocols as used to generate the cell atlas? In my opinion, testing with a dataset generated (optimally in another lab) with a different T. cruzi strain would truly be convincing to demonstrate that the described cell atlas can serve as a reference map.

Minor comments:

In the results section, line 79, different samples are described only with abbreviations (EP, SE/MT, ADT and so on). It is difficult to follow what they are when reading the manuscript for the first time. It would be easier when it was specified when the abbreviations are introduced for the first time.

What was the rationale of using SE/MT in bulk RNAseq (instead of purifying metacyclic trypomastigotes?). In the scRNAseq using SE/MT was justifiable to capture the transition stages, but in bulk RNAseq, it would be more interesting to obtain markers based on a population of more pure MTs.

References

Catta-Preta CMC, Ghosh K, Sacks DL, Ferreira TR. Single-cell atlas of Leishmania development in sandflies reveals the heterogeneity of transmitted parasites and their role in infection. Proc Natl Acad Sci U S A. 2024 Dec 24;121(52):e2406776121. doi: 10.1073/pnas.2406776121. Epub 2024 Dec 19. PMID: 39700146; PMCID: PMC11670217.

Reviewer #4

(Remarks to the Author)
I co-reviewed this manuscript with one of the reviewers who provided the listed reports. This is part of the Nature Communications initiative to facilitate training in peer review and to provide appropriate recognition for Early Career Researchers who co-review manuscripts.

Reviewer #5

(Remarks to the Author)
I co-reviewed this manuscript with one of the reviewers who provided the listed reports. This is part of the Nature Communications initiative to facilitate training in peer review and to provide appropriate recognition for Early Career Researchers who co-review manuscripts.

Version 1:

Reviewer comments:

Reviewer #2

(Remarks to the Author)
Overall, the authors have satisfactorily addressed all concerns. The revisions in response to all reviewers' comments have substantially improved the manuscript, and I recommend ACCEPTANCE.

As a minor point, reference 40 appears as '(!!! INVALID CITATION !!! 12)' and should be corrected.

Reviewer #3

(Remarks to the Author)
After carefully reviewing the revised version of the manuscript by Laidlaw et al., I note that although several of the comments and suggestions have been addressed, there is still a series of comments that remained unanswered. They are listed below.

1) The integration between bulk RNA-seq and scRNA-seq remains underdeveloped. In Figure 2d and Supplementary Fig. S5, the correspondence between scRNA-seq–derived marker genes and bulk RNA-seq expression profiles appears limited. At present, this comparison is largely qualitative. A formal statistical overlap test (for example hypergeometric or permutation-based) between top scRNA-seq markers and bulk RNA-seq differentially expressed genes, together with a

correlation analysis between bulk expression levels and scRNA-seq marker recovery, would help clarify whether single-cell markers simply reflect highly expressed bulk genes or capture additional biologically meaningful signals.

2) The doublet detection strategy is not fully convincing. While the authors argue that they avoided tools such as Scrublet or DoubletFinder to prevent removal of transitional states, filtering based on the number of expressed genes per cell poses a similar risk of excluding highly transcriptionally active or mixed-state cells. In addition, doublet rate estimates exist for related kinetoplastids (for example T. brucei in Briggs et al., 2021), and approximate rates can also be inferred from cell loading densities. Reporting expected doublet rates and indicating how many cells would be flagged by standard tools (even if not removed), would strengthen confidence that transitional clusters are not enriched for technical artifacts.

3) The fact that prediction scores are low for smaller clusters is exactly the problem here: classification performance is uneven and weakest for biologically relevant populations. While major life stages perform reasonably well (for example epimast_2 reaches ~0.85–0.87 F1 and meta_3 ~0.82–0.85), transitional and heterogeneous populations show substantially poorer performance. For example epi_meta_trans_7 drops to ~0.31–0.39, early_mid_amast_5 to ~0.43–0.47, and trypomast_9 to ~0.29–0.41 depending on the training fraction. These are precisely the populations most relevant for developmental interpretation, yet they are the least reliably classified. A weighted F1 score around 0.5 can therefore correspond to near-random classification of rare but biologically critical populations, while being dominated by performance on abundant epimastigote and trypomastigote populations. This strongly limits confidence in fine-grained atlas annotation and suggests that the model is primarily useful for coarse life-stage separation.

4) Evaluation metrics and generalization remain insufficiently addressed. Given the strong class imbalance between major life stages and rare transitional states, macro-averaged F1 scores and per-class performance summaries should be reported. Macro-averaged metrics would better reflect performance across all biological states rather than being dominated by abundant populations. It is also unclear whether any class weighting or rebalancing strategies were applied during training. Furthermore, the classifier is evaluated on data generated using the same strain, similar protocols, and the same laboratory environment as the training data, which further limits conclusions about robustness and generalizability.

5) The cell atlas was not validated either on a different strain or a dataset generated in a different lab, hence it is difficult to say how robust the data set it.

Reviewer #4

(Remarks to the Author)
I co-reviewed this manuscript with one of the reviewers who provided the listed reports. This is part of the Nature Communications initiative to facilitate training in peer review and to provide appropriate recognition for Early Career Researchers who co-review manuscripts.

Reviewer #5

(Remarks to the Author)
I co-reviewed this manuscript with one of the reviewers who provided the listed reports. This is part of the Nature Communications initiative to facilitate training in peer review and to provide appropriate recognition for Early Career Researchers who co-review manuscripts.

Version 2:

Reviewer comments:

Reviewer #3

(Remarks to the Author)
Overall, the authors have satisfactorily addressed most comments. I have no further remarks.

Open Access This Peer Review File is licensed under a Creative Commons Attribution 4.0 International License, which permits use, sharing, adaptation, distribution and reproduction in any medium or format, as long as you give appropriate credit to the original author(s) and the source, provide a link to the Creative Commons license, and indicate if changes were made.
In cases where reviewers are anonymous, credit should be given to 'Anonymous Referee' and the source.
The images or other third party material in this Peer Review File are included in the article's Creative Commons license, unless indicated otherwise in a credit line to the material. If material is not included in the article's Creative Commons license and your intended use is not permitted by statutory regulation or exceeds the permitted use, you will need to obtain permission directly from the copyright holder.
To view a copy of this license, visit https://creativecommons.org/licenses/by/4.0/

Response to reviewers

We would like to thank all the reviewers for their time and valuable comments on our manuscript. Addressing these has allowed us to substantially improve the manuscript. Please find our specific responses below.

REVIEWER COMMENTS

Reviewer #1 (Remarks to the Author):

This manuscript presents a comprehensive scRNA-seq atlas of the Trypanosoma cruzi life cycle, complemented by bulk RNA-seq data. The study offers detailed insights into gene expression dynamics across different life cycle stages and transitions, with a particular focus on the differentiation from epimastigote to metacyclic trypomastigote. The authors provide extensive UTR annotation and analyze multi-gene family expression at the single-cell level. Through trajectory analysis, they identify more than 900 genes associated with the epimastigote-to-metacyclic trypomastigote transition, including about 30 RNA-binding proteins. Strikingly, two distinct trypomastigote subpopulations emerge, one characterized by higher expression of metabolism-related genes and the other by elevated expression of surface protein genes (the latter being really surprising). Furthermore, single-cell analysis of multi-gene family expression reveals cell-specific repertoires of surface antigens in trypomastigotes.
This study represents a wonderful advance in our understanding of T. cruzi biology and is highly suitable for publication in Nature Communications. Below are some points for potential consideration, which I am confident the authors have already thought of:
1. The transition from metacyclic trypomastigote to amastigote warrants more discussion.

This is indeed an interesting point, and we carried out trajectory analysis for cell-derived trypomastigotes to intracellular amastigotes, but could unfortunately not identify a clear trajectory. Our experimental setup did not include infections with epimastigote-derived metacyclic trypomastigotes as for cost and complexity reasons we could not include this.

2. The implications of the observed alternative polyadenylation and antisense transcription patterns for gene regulation could be elaborated in greater depth.

Thank you for this suggestion. In the discussion we comment on alternative polyadenylation ("Our data shows that APA is common in *T. cruzi*, and as in other organisms it likely plays a key role in controlling mRNA stability and translation efficiency"). Rather than speculate further as to what the implications may be for *T. cruzi*, we have included two additional references that more extensively review the role of polyadenylation. Similarly, for the observation of antisense transcripts, we point out in the discussion that they may play a role in gene expression regulation, but beyond that we don't feel it is appropriate to hypothesize further. Instead, we envision that our dataset opens new avenues for other researchers to investigate the role of the antisense transcripts in *T. cruzi* gene regulation in more depth.

3. Investigating the functional significance of the two trypomastigote subpopulations

would be highly valuable.

Yes, we agree, but believe this is outside the scope of this manuscript, and hope that our data will stimulate other researchers to look into this further.

4. The potential regulatory roles of alternative polyadenylation sites and antisense transcripts merit experimental follow-up.

As above, we agree, but believe this is outside the scope of this manuscript, and hope that our data will stimulate other researchers to look into this further.

While addressing all these points would require extensive additional experimentation, **I do not believe such work should be requested as a prerequisite for publication.**
In conclusion, this manuscript presents an important and carefully executed study that substantially enhances our understanding of T. cruzi gene expression dynamics across its life cycle. The data and analyses provided offer a rich resource for future research. While some aspects of gene regulation and life cycle transitions remain to be fully elucidated, the current findings are already highly compelling. Pending very minor revisions, this work is well-suited for publication in Nature Communications.

We thank the reviewer to point out that those are suggestions rather than major points required for publication. These points very much illustrate how this new resource generates a range of hypotheses for further exploration.

Reviewer #2 (Remarks to the Author):

The authors generated and analyzed single-cell transcriptomic data of *Trypanosoma cruzi* using 10X Genomics technologies. This work represents one of the first single-cell transcriptomic datasets for this parasite and constitutes **a valuable contribution to the community studying *T. cruzi*.** Additionally, they provide an atlas that can be used to annotate life cycle stages and facilitate the annotation of UTRs. **The work is of significant general interest**; however, the manuscript requires substantial improvements in experimental design, and several important aspects of the data analysis need careful revision. Overall, we have observed that some paragraphs or sentences are incorrectly referenced, necessitating a thorough review. In the comments below, we will highlight some of these issues.

SAMPLES/DESIGN

To be considered an accurate single-cell atlas, other researchers should utilize a dataset as a reference. In this regard, the experimental design is critical. *Trypanosoma cruzi* shows significant variability depending on the clade/DTU used, which adds complexity to

the analysis. Moreover, its life cycle is more intricate than the traditional classification, divided into four stages: epimastigotes, amastigotes, metacyclic trypomastigotes, and cell-derived trypomastigotes. During its intracellular cycle, *T. cruzi* transitions through additional intermediate and clearly defined stages during trypomastigogenesis, including intracellular epimastigotes. In this context, several study aspects need substantial modification or clarification.

- Strain: It is necessary to specify that it belongs to DTU I. While this has the advantage of representing strains circulating in humans and wildlife, it also means that the results may not be extrapolated to other DTUs.

We have specified this now in the methods section (section 2.1): "*T. cruzi* parasites from the DTU1 strain Silvio (MHOM/BR/78/Silvio; clone X10/7) subclone A1 were used in this study[76]"

- Epimastigotes (EP): The parasite concentration in the 2-day cultures from which the samples were taken must be explicitly stated.

Due to the logistical complexity of the actual scRNA-seq experiments we did not count the parasites at point of harvesting. In satellite experiments, cell densities of 2-day and 10-day X10/7 epimastigote cultures were approximately 1.25×10^6 cells/ml and 2.6×10^7 cells/ml respectively. We have included this information in the methods section "2.2. Sample collection".

- Metacyclic trypomastigotes (SE/MT): Line 764 states that the sample was obtained from a 10-day-old culture. It is essential to clarify that the parasites were previously subjected to starvation and to provide data on the final parasite concentration. Concerning metacyclic trypomastigotes, we strongly suggest adding one more step: DEAE column enrichment, which typically yields populations with >95% MT.

For cell counts please see previous question. Regarding starvation, we have amended the text in the methods to say: "A mixture of stationary phase epimastigotes and metacyclic trypomastigotes (SE/MT) was obtained from a culture initiated with 3×10^5 epimastigotes/ml and maintained for 10 days without passaging, leading to starvation and induction of metacyclogenesis". Finally, with respect to DEAE column enrichment, our approach was to capture the heterogeneity of the culture, so we decided not to include a metacyclic trypomastigote enrichment step.

- ADT: It is essential to support this analysis with microscopy images of the three populations. Once trypomastigotes infect the host cell, two intracellular processes occur: amastigogenesis and trypomastigogenesis. Both processes involve intermediate and well-defined stages, with intracellular epimastigotes present before differentiating into trypomastigotes. Therefore, the term "ADT" should be revised to "CDT" (cell-derived trypomastigotes) to avoid the assumption that the preceding stage was amastigotes.

Thank you for these recommendations. We agree that microscopy images for the different populations provide valuable information and have now included images for all populations (Fig. S2). We have also changed ADT to CDT throughout the manuscript and figures.

Another critical point is the timing of CDT collection from the supernatant: these parasites should exit the cell no more than 12 hours earlier; otherwise, they begin transitioning into rounded forms known as extracellular amastigotes. Was this criterion taken into account? Can we ensure that the CDT population is greater than 90%? Again, microscopy images are essential.

Trypomastigotes were harvested seven days post infection, with a media change the evening before collection, so the majority of CDTs will have egressed less than 12 hours prior to isolation. We have clarified this in the methods, and now include microscopic images for this population, with Fig. S2G showing that the vast majority of trypomastigotes are elongated, with no rounded forms visible.

- AMA: The progression of the intracellular cycle varies depending on the strain, cell type, and MOI. The authors used two MOIs (10 and 1.5) to obtain AMA samples, presumably to maintain the 120-hour time point. However, this raises the question of which cellular form is actually being reported in the single-cell data. The authors rely on the work of Li et al., but that study used the Y strain belonging to DTU II, whose cycle timing differs from that of DTU
I. In addition, intracellular epimastigotes precede the differentiation to trypomastigotes (see below). Therefore, returning to the fundamental issue, it is crucial to document the cellular forms used in sample collection with images. Otherwise, this study cannot be considered an atlas.

Images for all populations are now provided in Fig. S2.

INTRODUCTION

This overly long introduction focuses too much on very general and well-known aspects of Chagas disease and its cycle. The life cycle explanation is overly detailed and could be summarized and modified to introduce the concept that, in addition to these four stages, there are additional and transitional stages (in particular, the intracellular epimastigotes constitute a defined stage), which also vary between clades or DTUs.

Articles that may contribute to revising the cycle:

- https://doi.org/10.1016/0014-4894(78)90159-5
- https://doi.org/10.1007/BF00931508
- https://doi.org/10.1006/expr.1999.4423
- https://doi.org/10.3390/pathogens13100866
- https://doi.org/10.1186/s13071-018-2664-4

Thank you for this comment. We have shortened the introduction by ~100 words and refer now to the intracellular epimastigotes. We have also included some of the references you kindly provided.

Note that the last paper is reference 4 in the main manuscript. Although it is already cited, it is incorrectly referenced at the end of the sentence:

"Differentiation into non-motile, replicative amastigotes starts in parasitophorous vacuoles,

followed by escape to the host cell cytosol, where differentiation is completed."

There are two errors here:

1. The cited article refers to metacyclogenesis, which means the cycle in the vector, while the authors discuss the intracellular cycle in mammals.

We are not completely sure what the reviewer is referring to here, reference 4 in the original submission was: Batista, et al. The Parasitic Intracellular Lifestyle of Trypanosomatids: Parasitophorous Vacuole Development and Survival. Front Cell Dev Biol 8, 396, doi:10.3389/fcell.2020.00396 (2020), whereas the last paper in the reviewer's list is: Gonçalves et al. Revisiting the *Trypanosoma cruzi* metacyclogenesis: morphological and ultrastructural analyses during cell differentiation. Parasites Vectors 11, 83 (2018). Our reference 4 seems correct to us as it covers the intracellular cycle.

2. Amastigotes are not "non-motile" (https://doi.org/10.1128/mbio.03556-22).

The authors in this reference (https://doi.org/10.1128/mbio.03556-22) state in their conclusion: "this work has provided the first description of flagellar beating in the **nonmotile** intracellular amastigote stage of *T. cruzi*.". Our understanding from this work is that, while they indeed detect flagellar beating in amastigotes, they do not suggest this leads to amastigote motility. Instead the authors suggest various other possible roles for flagellar beating. While in this context we believe it is still correct to say that amastigotes are non-motile, as part of shortening this section we have removed "non-motile".

RESULTS

The reasoning for the authors' choice of pools for sequencing is unclear in the text and must be explained.

Thank you for this comment. These experiments are expensive and it was important for us to balance cost with ability to assign life-cycle stages directly. This is standard practice in many similar experiments. HTO Cite-Seq could be utilised, however, this was not possible in our setup. However, we were able to split the samples and have now provided the following additional explanation for the pooling strategy in the methods section:
"In order to balance cost with ability to assign parasites to a specific life cycle stage some samples were combined in equal proportions for scRNA-seq processing; AMA120h and EP were pooled as we expected to see clearly distinct populations for these two samples and AMA6h and AMA24h were pooled as we sought to maximise the heterogeneity at early amastigote timepoints without need to assign cells directly to the 6h or 24h post infection populations. Each pool contained 16,500 cells."

We are deeply concerned about several aspects of the data analysis that require careful revision and reanalysis. As is well known, the genome of *T. cruzi* is highly repetitive; approximately 50% of the genome consists of repetitive sequences, including multigene families, repetitive elements, and transposable retroelements, among others. This genomic characteristic has posed significant challenges for scientists working with this parasite, who have extensively sought effective strategies to ensure accurate data interpretation and reliable results. A crucial part of the transcriptome analyses of *T. cruzi* is the treatment of

multimapping reads. Excluding multimapping reads from the analysis oversimplifies biological reality. Evidence suggests that this is not a correct strategy. In this study, the authors conduct expression analysis of multigene families for which, by their very nature, most of the reads generated by their transcripts are multimapping. This issue becomes even more critical, highlighting a significant aspect that requires review. The authors should re-evaluate and interpret the data, considering these factors.

The reviewer brings up a salient point with respect to dealing with, and interpreting, multi-mapping reads from kinetoplastid RNA-seq data. First, we would like to state that the “core genes” that we describe in our manuscript are not repetitive. The repetitive portion of the genomes is relevant for the multi-gene family analysis (Figure 3e and Figs. S6 – S8). Our approach does indeed simplify the biological reality for those repetitive gene families, however, >95% of all members of the multi-gene families analysed (TS, DGF-1, GP63, MASP, mucin, RHS) provided UMI counts in our analysis, showing that differences in the 3’UTR sequences allow us to measure expression for the vast majority of repetitive genes.

Further, we would argue that including or excluding multi-mapped reads each comes with its own unique set of challenges. With our approach (i.e, not including the multi-mapped reads), we are deflating the UMI counts of repetitive genes, which is more conservative and leads to them having a lesser impact on the variation in the dataset. By including the multi-mapped reads there is the opposite issue, where one inflates the importance of the repetitive genes, as it is uncertain of which gene the UMI comes from.

Interestingly, and importantly, both approaches ultimately seem to reveal the same underlying *T. cruzi* biology, with Inchausti et al., who included multimapping reads, coming to the same biological conclusions as us in their preprint https://elifesciences.org/reviewed-preprints/105822v1, providing added validity to our results.

- Line 79: the authors should not use abbreviations the first time they describe the samples (e.g., EP, SE, MT, AMA).

We now introduce the abbreviations the first time they are mentioned.

- Figure 1 illustrates the samples used in the study but does not present any results. Since the parasite's life cycle is already well-documented in the research community, creating a detailed schematic is unnecessary. Instead, a brief description of the sample collection process in the methods section would be sufficient. The figure should be moved to the supplementary material section.

We have moved figure 1 to the supplementary information section.

- Figure 2. How do the authors explain the differences in the SE/MT replicates? In replicate 2, there appears to be a cluster of cells not present in replicate 1.

The cluster the reviewer refers to appears to be present in both replicates, but indeed the number of cells in this cluster in sample SE/MT1 is much lower than in SE/MT2. We can’t offer an explanation beyond this being biological variability, in spite of our best efforts to maintain identical conditions for both replicates.

- UTR mapping and Figure 4. There is a missing citation and inclusion of an important study of UTR genome-wide annotation conducted on *T cruzi.* (https://doi.org/10.3389/fgene.2018.00671). The authors list several results that cannot be attributed to the only two figures they present in this section. They describe results from weak analyses (based on inspections of graphs but which are not shown). Further studies and quantifications to support the results described in the "UTR mapping" section are needed to substantiate these results. The authors must provide more evidence and information about the described results (lists of the "missing transcripts," methods, and criteria they used to say there is an unannotated transcript, the possibility that the "unannotated" genes are long ncRNAs).

  We are not sure if we can completely follow the reviewer here. First, the figures in question are in supplemental figure 2a, which show our manual findings (Fig S9 in revised supplementary information). I would also respectfully disagree that manual inspections are weak results – rather we want to highlight with our examples that scRNA-Seq and bulk RNA-seq can be used to refine and improve annotation. So the focus of our resource is not the exact number of new genes, but the fact that our data allow the community to explore different aspects of Trypanosoma biology.

  However, to help with the readability we adapted the section and added additional panels to the main UTR figure (now Figure 5), with additional information in Fig. S9. The suggested reference has now also been added to the UTR results section.

  Overall our aim was not to provide an improved annotation, but rather show that our data can be used by others to improve existing annotations.

- Figure 4. The authors list several results from which no merits can be derived from the figure. They describe results from weak analysis based on inspections of graph, but they do not appear. Moreover, in T. cruzi, genes lack introns except for a few distinct exceptions; thus, referring to the coding portion of a gene as an exon is incorrect. Finally, as mentioned above, the sentence "Our analysis estimates that there are ~1,500 missing genes in the DM28c annotation" lacks foundation. What is the list? Or at least the location? We strongly recommend further argumentation and substantiation of these statements throughout the text.

Thank you for these comments, we have changed exon to CDS (coding sequence) in the figure and have decided to remove the statement around the ~1,500 missing genes, as a new assembly of the Dm28c genome has become available on BioRxiv. This latest assembly is a fantastic reference with 14 complete chromosomes. As annotation of this new reference is ongoing (personal communication) we think there is no value for us to discuss potentially missing genes from the older assembly available on TriTrypDB.

- This is not the first time the transcription of both strands has been described in *T. cruzi*. This was very well documented in 2021 (https://doi.org/10.1371/journal.ppat.1009254). The authors should consider and include previous studies

conducted on this parasite throughout the entire manuscript.

We have now included this reference in the discussion: “Our RNA-seq data confirms the previously reported presence of antisense transcripts in T. cruzi[73], and in addition our single-cell data reveals reverse transcripts at the 3’UTR end, in the opposite direction of the main transcript. While the role of these transcripts in T. cruzi remains unclear, antisense transcripts have been shown to play a role in gene expression regulation in other trypanosomes[74-76].” The novelty here is the different types of reverse transcripts seen in bulk RNA-seq and single-cell RNA seq.

- Can these results be validated in other transcriptomes? To ensure consistency in the analysis, the scRNA-seq and bulk transcriptome results should be compared with the transcriptome published in 2023 (DOI: 10.1038/s41564-023-01483-y), which includes a deep sequencing analysis of three parasite stages from a DTU I strain (Dm28c used in this work for analysis). The comparison must focus mainly on marker genes to validate them as markers. Additionally, a comparison with the transcriptome from Li et al. should be performed to assess whether these markers apply to a strain from a different DTU (II in this case). With these comparisons, authors could establish whether the detected genes are definitively stage markers.

Our submission included a comparison with markers described in five previous *T. cruzi* transcriptomics papers, that all include at least 3 parasite stages (Supplementary File 2). We did not include the paper you refer to here (DOI: 10.1038/s41564-023-01483-y) in this analysis as this paper does not seem to report any marker genes.

- Related to the previous point, it has been recently published a preprint (https://doi.org/10.1101/2025.01.14.633000) entitled “Single-cell RNA-seq reveals trans-sialidase-like superfamily gene expression heterogeneity in Trypanosoma cruzi populations.” Authors perform single-cell RNA sequencing of T cruzi amastigotes and trypomastigotes and determine markers from each stage. It is essential to compare the markers. Note that the authors YES consider the multimapping reads in the referred preprint. It would be enriching to compare in the discussion the results of that other study in which there are some similar findings. It would be even more compelling to incorporate the results of an independent analysis that reaches similar conclusions.

3.

This paper was put onto bioRxv after we submitted this manuscript to Nature Communications, but we have now cited this paper in our discussion of the trypomastigote results. It is indeed reassuring that both sets of results validate each other, i.e. there exists transcriptionally distinct trypomastigote subsets, and individual cells have unique surface protein repertoires, as well as each providing complementary information. Furthermore, as the other paper is more focused on gene families, which will be more repetitive, it makes sense for them to consider multi-mapped reads. As we are more focused on the lifecycle at large, it made more sense (in our minds) to only look at single mapping reads. We have included a comment in the discussion on how both multimapping and non-multimapping approaches yield the same conclusion.

- Line 862. The authors must specify the units to define the threshold, as "10" alone is meaningless.

We thank the reviewers for pointing out this lack of proper definition. We have now updated the text to read “...the genes whose read counts were less than 10 in any 11 of our 12 samples...”

- The authors describe only ONE gene for the amastigotes stages to indicate that the bulk RNA-seq validates the scRNA-seq atlas. However, the biology of *T. cruzi* is too complex to be reduced to discussing a single gene. We expect a more comprehensive analysis to enrich this section.

We are uncertain if we understand the reviewer's comment fully, as in figure 2D (new figure numbers) we provide five markers for both the late_amast_1 and early_mid_amast_5 clusters and their expression in the bulk RNA-seq data. Most of these markers show high expression in the amastigote bulk RNA-seq datasets, thus validating the results of the scRNA-seq data.

- Figure 3d: It would be interesting to know the results of the reverse analysis: how is the expression in single-cell RNA-seq of marker genes identified in bulk RNA-seq?

We agree with the reviewer that this is an interesting point of comparison and have now included this in the paper as supplementary figure 5. When we ran this analysis, the results show that some bulk RNA-seq marker genes are expressed well in the scRNA-seq data, although many are not. As 10X scRNA-seq only captures the 3’ end of the transcript, while bulk RNA-seq is full length, this may explain the lack of expression of bulk RNA-seq markers in the scRNA-seq data. However, scRNA-seq's greatest strength is being able to capture hetereogeneity within lifecycle stages, which we have demonstrated in the paper.

- Line 840: “The 3’ UTRs annotations of the Trypanosoma cruzi Dm28c 2018 (73) were extended by…” Another mistake in the references. Reference 73 is a “Genome Announcement” from 2014, whereas Dm28c_2018, as its name suggests, was communicated in 2018.

Thank you very much for picking up on this error, we have corrected this.

-
- 211-213: “This analysis allowed us to identify highly specific marker genes for (…) of these in line with previously published results.” Add the references for the previously published results.

The references were included in Supplementary File 2, but we agree that we should show them in the main manuscript too, so they have now been added.

Reviewer #3 (Remarks to the Author):

The authors present a well-executed study using single-cell transcriptomics to analyze the different life stages of Trypanosoma cruzi. This is a first single-cell transcriptomic data set for the protozoan parasite T. cruzi and will certainly provide a valuable resource for the research community. The manuscript presents a single-cell atlas for in-vitro grown parasites, allowing identification of clusters of epimastigotes, metacyclic trypomastigotes, transitionary stages between epimastigotes and metacyclic trypomastigotes, amastigote-derived trypomastigotes, and amastigotes at different stage of infection. It provides novel insights into the heterogeneity of different life stages by detecting transcriptionally different populations of trypomastigotes and describing different cell-specific multi-gene family repertoires. In addition, it presents a bulk RNAseq dataset with markers of the in vitro-derived life stages studied here. Finally, by combining scRNAseq and bulk RNAsed data it maps UTRs, it identifies alternative polyadenylation sites, and reverse strand and antisense transcripts.

General comments

One of the main deliverables of the manuscript is the generation of the complete T. cruzi cell atlas, while actually not all transitions between life stages, and potentially not all the life stages are being described. The authors focus their trajectory analysis solely on the transition between epimastigotes and metacyclics, and the transition from the metacyclic trypomastigote to amastigote is missing. However, if the single-cell RNA-seq dataset captures all life stages, a full trajectory analysis should, in principle, allow for the reconstruction of all developmental transitions. As such the cell atlas cannot be called “complete”. In addition, dormant amastigotes seem to be missing, even though their existence and their potential role for the development of novel therapeutics is mentioned by the authors in the introduction. It is not clear whether “late_amast” could be considered dormant or dormant amastigotes were not detected in the dataset?

The reviewer is correct that we have not captured all the life cycle transitions, but we nevertheless cover the majority of known life cycle stages. In view of this we still believe that the term “atlas” is appropriate but agree that we should remove “complete” (now removed from the abstract). As for the dormant amastigotes, we could not detect these, potentially because transcript levels in this population are likely low, and 10x is not the most suitable method for such cells.

Overall, the manuscript focuses on annotating the main life stages in the single-cell atlas, and confirming the correctness of the annotation, but it does not provide novel insights into biology of T. cruzi. There is no experimental validation of the findings: no novel life-stage specific markers are experimentally validated or no functional verification of newly described genes regulating the transition from epimastigotes to metacyclis has been done. Also, no evident novel biological hypothesis based on the single cell transcriptome findings are presented here.

First, we would like to highlight that this is a resource paper, and that it is not in the scope of this work to carry out experimental validation of new findings, instead we believe our work will support other researchers in the field, and aid in the generation of testable hypotheses. We do however present new biological hypotheses, a few examples are: the existence of two transcriptionally distinct trypomastigote populations which may align with previously characterized morphologically and functionally different populations, the presence of cell-

specific surface-protein repertoires and a potential role in immune-evasion, and the identification of multiple RNA-binding proteins that may have a role in epimastigote to metacyclic trypomastigote differentiation.

Metacyclic trypomastigotes and trajectory analysis: in Fig 2a, two populations of metacyclic trypomastigotes (meta_8 and meta_3) are detected and the markers associated with these two clusters are presented in supplementary file 1. Then, later in the manuscript a trajectory analysis of the transition from epimastigotes to metacyclics is presented. It is not clear how these two populations of metacyclics are linked to the trajectory analysis. Would one population be early metacyclics and the other one late? Are they functionally different? Are they both non-replicative? Recent paper in Leishmania described two populations of metacyclics with early ones surprisingly being replicative, and the late ones not (Catta-Preta et al., 2024). Is a similar scenario possible here?

When we focused on the metacyclic, epimastigote and transitionary clusters, the meta_8 population appeared distinct from the rest of the cells and was thus excluded (covered in Methods section). The reviewer brings up an interesting point in terms of replicative vs non-replicate metacyclics. Early in our analysis, we were interested in seeing if cell cycle was captured in the transcriptomic data, as previously published analyses of *T. brucei* and *Leishmania* showed that it was in these contexts. We however did not pursue this investigation far, as the regulation of the cell cycle in *T. cruzi* is shown to be more at the translatome and proteome level, rather than transcriptome (Chavez et al., 2021).

While the bioinformatics analyses presented are of high quality, some methodological decisions are not always clearly justified. Additionally, certain key details are missing from the supplementary tables.

• Doublet Detection: The authors use the number of genes expressed as an indicator of doublets. However, this is a rather rudimentary approach, given that more sophisticated methods, such as Scrublet, are available. This method also risks excluding highly transcriptionally active cells while failing to filter out some true doublets. Furthermore, considering the variability in the number of genes expressed across different samples, does applying a fixed threshold make sense, or should this criterion be adjusted per sample? Since some samples contain a mix of human and T. cruzi cells, a more precise estimate of doublet occurrence should be possible, allowing for a more data-driven threshold in doublet detection.

The reviewer is correct that there are more sophisticated methods for doublet detection. We decided not to use these methods for two main reasons. 1: as we are interested in transitions between the lifecycle stages, which may have transcriptomic profiles that are a mix of lifecycle stages, we did not want them to be removed as “hetereotypic” doublets. 2: These methods are best applied when the doublet rate is known a priori. For mammalian samples this is readily available, but the rate in *T. cruzi* is not known. Individual thresholds were calculated for each sample as detailed in supplementary table 4.

• Supplementary Table 1: While the number of cells loaded onto the 10X platform is reported, it would also be valuable to include the number of cells recovered for each dataset. Additionally, the number of UMIs per cell after filtering is not mentioned in any supplementary table.

We agree with the reviewer that this is a good addition. We have added the number of *T. cruzi* cells recovered for each 10X run to supplementary table 3.

• Supplementary Table 3: Different cut-offs were applied depending on the sample analyzed. While this approach is reasonable, it raises the concern that clustering may primarily reflect differences in transcriptional activity or read depth per cell rather than true gene-level transcriptional differences.

We use the normalisation method recommended by Ahlmann-Eltze & Huber 2023 to account for read depth differences, meaning the clustering should not be driven by transcriptional activity or read depth

• Supplementary Table 4: The rationale for imposing a maximum threshold on the number of reads mapping to T. cruzi is unclear. In samples consisting solely of T. cruzi, one would expect the fraction of reads mapping to its genome to be as close to 100% as possible. Could the authors clarify this decision?

We thank the reviewer for spotting this mistake. The column name should read “% reads mapping to *T. cruzi* mitochondrial genes maximum”. We have changed this in supplementary table 4

The 3' UTR prediction based on the transcriptome data presented in this study is a valuable effort. However, it is unclear why the authors used a relatively rough approach—defining the 3' UTR region by extending 2,500 bp beyond the gene's end position—for the scRNA-seq analysis when a high-quality list of 3' UTR regions is made available within their work. Could they clarify the reasoning behind this choice?

This is a fair point. All our “atlas” work was carried out with the 2,500 bp boundaries before we carried out the UTR analysis. In the new version of the manuscript we have now included a section comparing the data generated using 2,500 bp boundaries with the data using the new list of 3’ UTRs. This is covered in the final section of the results and shows that overall expression patterns are similar between both mappings with the trajectory for metacyclogenesis showing similarity and the trypomastigote subsets, with different trans-sialidase expression, also detected. We believe that this comparison will be of interest to the readers (rather than removing the 2,500bp mapping).

The added value of the bulk RNA-seq data in this study is not entirely clear. The authors appear to place significant emphasis on previously identified biomarkers rather than using the bulk RNA-seq results to derive novel insights. To better justify the integration of bulk RNA-seq with scRNA-seq, the authors should provide additional details regarding the overlap between scRNA-seq marker genes and differentially expressed genes from bulk RNA-seq. Specifically, they should assess whether this overlap is statistically significant (e.g., using a hypergeometric test). Furthermore, it would be informative to visualize the top expressed genes from bulk RNA-seq on the scRNA-seq UMAP projections to determine how well bulk-dominant genes map onto single-cell clusters. Additionally, the authors should explore the relationship between bulk RNA-seq expression levels and scRNA-seq marker recovery—do the scRNA-seq markers predominantly represent highly expressed genes from bulk RNA-seq, or does scRNA-seq also capture lowly expressed genes that might be biologically relevant?
Moreover, already 5 papers with bulk RNAseq of different T. cruzi life stages are already published (and are mentioned in the supplementary file 2), hence the novelty of and the need for the current bulk RNAseq datset is not evident.

The primary purpose of the bulk RNA-seq dataset was not novelty per se, but to support validation of the clusters we identified in our scRNA-seq data. For example, we wanted to know whether the marker genes we identified in the scRNA-seq clusters have the highest

expression in the equivalent bulk RNA-seq datasets, which we carry out in figure 2d. While there are indeed numerous bulk RNA-seq papers (which we draw on in this paper for insight), we wanted paired scRNAseq and bulk as this is a resource paper. Furthermore, the bulk RNA-seq papers published sometimes use different strains of *T. cruzi* or do not profile all the lifecycle stages, which makes our bulk RNA-seq data more comparable to our scRNA-seq data. We have provided a visualization of the expression of bulk RNA-seq marker genes on the scRNA-seq data in supplementary figure 5.

scPoli predictions: While the authors clearly describe their approach to training and testing the life-stage classifier, the reported results are less convincing. The average F1 score occasionally drops to 0.60, and for certain life stages, it falls as low as 0.30—values that are considerably lower than the ≥0.80 F1-score typically expected in machine learning evaluations. Moreover, the classifier is validated on a sample generated using the same experimental setup as the training data. This raises concerns about its generalizability—how well it would perform in more distinct settings, such as different species or alternative single-cell platforms. To better assess performance, presenting the confusion matrix would be valuable. Additionally, do the authors have insights into the key features driving the classification? It remains unclear whether the classifier is leveraging biologically meaningful gene expression signatures or if it is primarily responding to overall transcriptional activity, which could reduce its ability to distinguish life stages robustly.

The reviewers are right that the prediction scores can be quite low, however this mainly impacts smaller clusters (like the epi_meta_trans_7 cluster) and when only a small subset (i.e. 10%) of the data is used to train the classifier. Furthermore, the scPoli paper results have the weighted average for F1 score varying between ~0.95 and ~0.5 depending on dataset they tested, putting us within the expected range. As scPoli uses variational autoencoders, it's difficult to say exactly what features are important in terms of the classification. It is of course a worry that the autoencoder is not relying on biologically meaningful information, but as the authors of scPoli have applied the method to various human datasets and observed good results, it gives us confidence that a similar result is seen for *T. cruzi.*

The lack of a confusion matrix was an oversight on our part. We have now included the confusion matrices as a supplementary figure (Fig. S4).

Validation of the cell atlas as a reference map for other datasets: it is not clear what kind of sample was used to validate the atlas. Was it generated using the same strain with exactly the same protocols as used to generate the cell atlas? In my opinion, testing with a dataset generated (optimally in another lab) with a different T. cruzi strain would truly be convincing to demonstrate that the described cell atlas can serve as a reference map.

We agree that the ideal scenario would be to use results from different strains generated in a different lab to test the mapping capabilities of the atlas. As this was not possible, in part due to the lack of published scRNA-seq *T. cruzi* data (the data from Inchausti et al. is not publicly available), we opted for a sample that was generated more than one year before the rest of the dataset and from amastigotes derived from a different timepoint post-infection to get as close to a different dataset as possible from the ones contained in the atlas. The strain was the same, and the protocol very similar.

Minor comments:

In the results section, line 79, different samples are described only with abbreviations (EP,

SE/MT, ADT and so on). It is difficult to follow what they are when reading the manuscript for the first time. It would be easier when it was specified when the abbreviations are introduced for the first time.

Absolutely, we have now explained the abbreviations when they are introduced.

What was the rationale of using SE/MT in bulk RNAseq (instead of purifying metacyclic trypomastigotes?). In the scRNAseq using SE/MT was justifiable to capture the transition stages, but in bulk RNAseq, it would be more interesting to obtain markers based on a population of more pure MTs.

Our rationale was to use exactly the same populations for both bulk and scRNA-seq experiments, but you present a fair point. Unfortunately, we lack the resources to repeat this experiment with MT purification, but we believe that ultimately this does not affect the key results presented in the manuscript (i.e. the single cell data), and the bulk RNA-seq data as generated still allows validation of the scRNA-seq dataset.

Reviewer #4 (Remarks to the Author):

I co-reviewed this manuscript with one of the reviewers who provided the listed reports. This is part of the Nature Communications initiative to facilitate training in peer review and to provide appropriate recognition for Early Career Researchers who co-review manuscripts.

Reviewer #5 (Remarks to the Author):

I co-reviewed this manuscript with one of the reviewers who provided the listed reports. This is part of the Nature Communications initiative to facilitate training in peer review and to provide appropriate recognition for Early Career Researchers who co-review manuscripts.

Please find below our point-by-point responses to Reviewer #3’s comments.

1) The integration between bulk RNA-seq and scRNA-seq remains underdeveloped. In Figure 2d and Supplementary Fig. S5, the correspondence between scRNA-seq–derived marker genes and bulk RNA-seq expression profiles appears limited. At present, this comparison is largely qualitative. A formal statistical overlap test (for example hypergeometric or permutation-based) between top scRNA-seq markers and bulk RNA-seq differentially expressed genes, together with a correlation analysis between bulk expression levels and scRNA-seq marker recovery, would help clarify whether single-cell markers simply reflect highly expressed bulk genes or capture additional biologically meaningful signals.

Answer:

To address this we have now statistically tested the overlap of the marker genes from the scRNA-seq clusters and the highly specific bulk RNA-seq marker genes using a hypergeometric test. These results further validate our cluster labels assignments, with the cluster markers mostly significantly overlapping with bulk RNA-seq marker genes from their equivalent lifecycle stage, e.g. the meta_3 marker genes only significantly overlap with the bulk RNA-seq metacyclic marker genes. This information is now included in supplementary figure 7 and in the “Bulk RNA-seq across all life cycle stages validates the scRNA-seq atlas” results section.

In response to the reviewer’s comments about whether the scRNA-seq cluster marker genes represent highly expressed bulk genes, when looking at a plot of the log1p transformed gene counts annotated with whether a gene was found to be a scRNA-seq marker gene (see plot below), the marker genes identified through scRNA-seq overall are among the higher expressed genes in the bulk RNA, but there nevertheless is a spread of expression indicating that scRNA-seq is not just capturing the most highly expressed bulk genes. Furthermore, the power of scRNA-seq, in comparison to bulk RNA-seq, is not that it can reveal marker genes of established cell types, but that it can reveal heterogeneity within stages, as we have demonstrated in our comparisons of trypomastigote heterogeneity and investigation of metacyclogenesis.

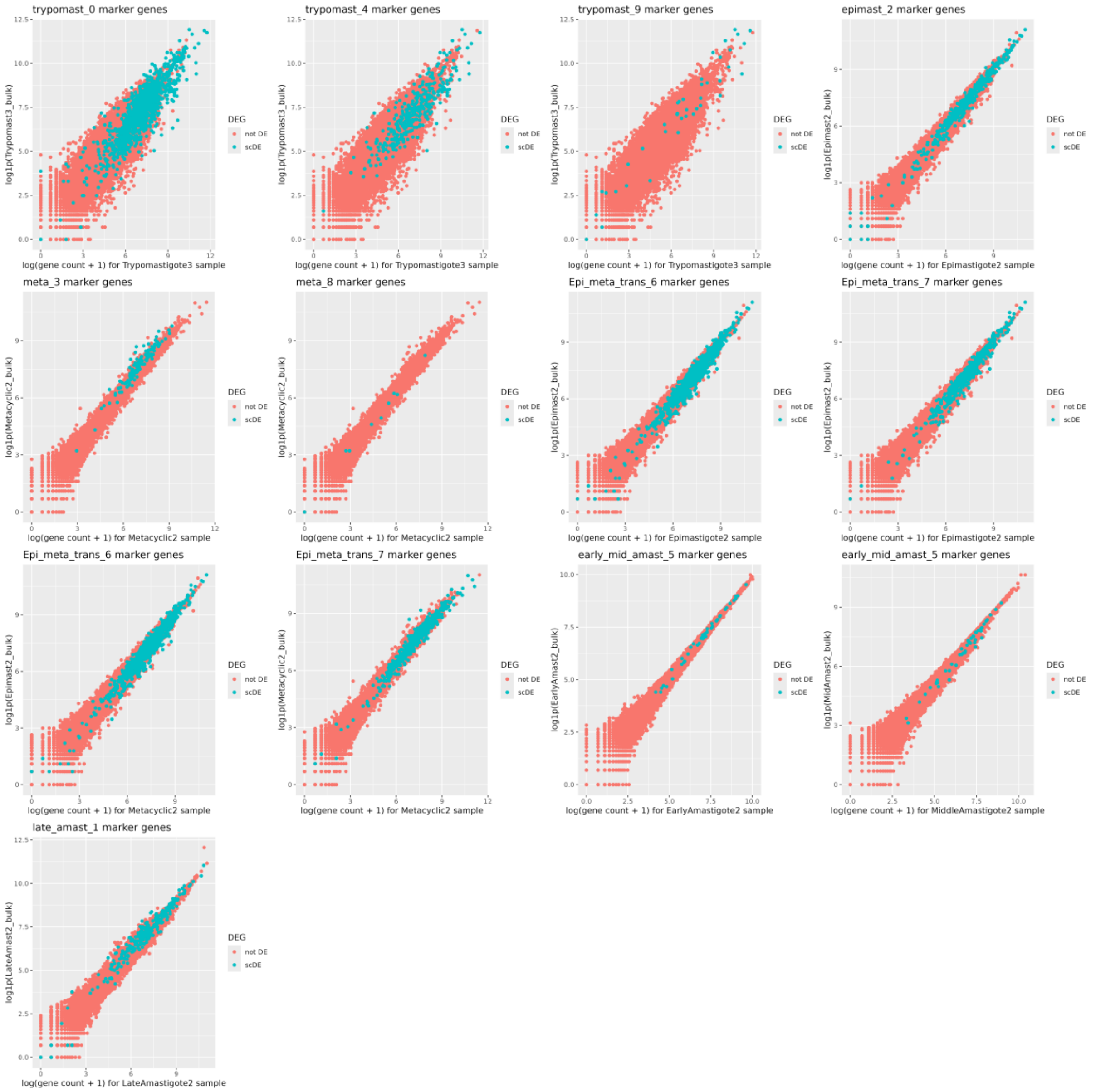

Dot plots of bulk RNA-seq biological replicates showing the log(gene counts +1) for the bulk RNA-seq data where each gene is coloured by whether the gene was identified as a marker gene of different scRNA-seq clusters.

2) The doublet detection strategy is not fully convincing. While the authors argue that they avoided tools such as Scrublet or DoubletFinder to prevent removal of transitional states, filtering based on the number of expressed genes per cell poses a similar risk of excluding highly transcriptionally active or mixed-state cells. In addition, doublet rate estimates exist for related kinetoplastids (for example T. brucei in Briggs et al., 2021), and approximate rates can also be inferred from cell loading densities. Reporting expected doublet rates and indicating how many cells would be flagged by standard tools (even if not removed), would strengthen confidence that transitional clusters are not enriched for technical artifacts.

Answer:

We agree with the reviewer that filtering based on number of genes expressed is also not optimal, however our approach is similar to others when analysing kinetoplastid scRNA-seq data (e.g. Briggs et al., 2021, Briggs et al., 2023, Louradour et al., 2022, Catta-Preta et al., 2024 & Inchausti et al., 2025). Furthermore, the estimated doublet rate the reviewer refers to from Briggs et al., 2021 is a cross-species doublet rate, not the total doublet rate for T. brucei or L. mexicana individually.

To address the reviewer's point, we applied Scrublet with an estimated doublet rate of 10% for the two lifecycleMix batches and 8% for the others (higher than the expected doublet rate for the number of cells loaded for these batches) to all our scRNA-seq batches and checked if any of our scRNA-seq clusters were particularly enriched in terms of doublets. If one cluster was specifically enriched for doublets across our batches, then this may indicate that this is not a biologically relevant cluster, but rather represents doublets. In the case the reviewer is referring to, if the epi_meta_trans_6 and epi_meta_trans_7 clusters are enriched for doublets across most of the batches, this could suggest they are not transitional, but rather epimastigote and metacyclic doublets. The results (which are now in the manuscript as Supplementary Figure 4) show that no cluster is particularly enriched for doublets across the batches, suggesting that our clusters do represent biologically relevant cells.

References:
Briggs et al., 2021 DOI: 10.1038/s41467-021-25607-2
Briggs et al., 2023 DOI: 10.7554/eLife.86325
Louradour et al., 2022 DOI: 10.7554/eLife.73488
Catta-Preta et al., 2024 DOI: 10.1073/pnas.2406776121
Inchausti et al., 2025, reviewed preprint: https://doi.org/10.7554/eLife.105822.1

3) The fact that prediction scores are low for smaller clusters is exactly the problem here: classification performance is uneven and weakest for biologically relevant populations. While major life stages perform reasonably well (for example epimast_2 reaches ~0.85–0.87 F1 and meta_3 ~0.82–0.85), transitional and heterogeneous populations show substantially poorer performance. For example, epi_meta_trans_7 drops to ~0.31–0.39, early_mid_amast_5 to ~0.43–0.47, and trypomast_9 to ~0.29–0.41 depending on the training fraction. These are precisely the populations most relevant for developmental interpretation, yet they are the least reliably classified. A weighted F1 score around 0.5 can therefore correspond to near-random classification of rare but biologically critical populations, while being dominated by performance on abundant epimastigote and trypomastigote populations. This strongly limits confidence in fine-grained atlas annotation and suggests that the model is primarily useful for coarse life-stage separation.

Answer:

We realise that due to poor labelling of the Supplementary Table 5, we may have caused confusion around the results. The quoted F1 scores for the epi_meta_trans_7, early_mid_amast and trypomast_9 were results when 10% of the atlas was used to train scPoli to predict the remaining 90% of the atlas. When

training a model, it would not be common practice to use such a small subset of the dataset, this table was just to show the extremes of performance depending on the training fraction. For example, epi_meta_trans_7 shows substantially higher F1 scores (up to 0.7) when a larger fraction of cells is used. We have amended the table to improve clarity. Furthermore, the performance for trypomast_0 and trypomast_4 populations, which are both fine-grained and biologically relevant, are stable. As the focus of this paper is providing a biological resource, rather than testing the limits of a prediction model, we have removed the columns for the model performance when 10% of the atlas was used for training, as it represents an extreme case which would be seldom carried out in practice.

While the fact that F1 scores represent a harmonic mean of recall and precision means that interpreting them for less abundant classes can be biased, we have also included the confusion matrices (as suggested by previous reviewer comments) in Supplementary Figure 5 for these experiments so readers can see the raw data themselves. We believe that the F1 scores give a good indication of the results of the cell type label prediction, with this being what the original scPoli paper uses to assess performance.

4) Evaluation metrics and generalization remain insufficiently addressed. Given the strong class imbalance between major life stages and rare transitional states, macro-averaged F1 scores and per-class performance summaries should be reported. Macro-averaged metrics would better reflect performance across all biological states rather than being dominated by abundant populations. It is also unclear whether any class weighting or rebalancing strategies were applied during training. Furthermore, the classifier is evaluated on data generated using the same strain, similar protocols, and the same laboratory environment as the training data, which further limits conclusions about robustness and generalizability.

Answer:

The reviewer makes a good point that the weighted average F1-scores are biased by class imbalance, as in this atlas. For Fig. 1f we have now changed the scores from weighted average F1-scores to macro-averaged F1 scores, which does not substantially affect the results. As noted in the previous comment, we have provided the confusion matrices so readers can more easily review the raw prediction results.

No class weighting or rebalancing strategies were utilised during the training of the models. We have now explicitly stated this at the end of section 2.6.8 of the Materials & Methods.

With respect to the last section of comment 4 and the whole of comment 5, the lack of validation on a dataset from a different strain/lab environment is indeed a limitation. However, it was outside the scope of our work to test robustness across different strains, protocols, and laboratory settings. We are confident to have shown that our data reflects the main lifecycle stages, as well as specific subsets of lifecycle stages of *T. cruzi*; which is the purpose of the atlas. How well this reflects other scRNA-seq experiments on *T. cruzi* is something we look forward to reading future

papers from researchers in the *T. cruzi* field who utilise this atlas to compare and contrast their data with ours.

5) The cell atlas was not validated either on a different strain or a dataset generated in a different lab, hence it is difficult to say how robust the data set it.

Answer:

Please see the last paragraph of the response to comment 4

ROUND 1 REVIEWER 2 ATTACHMENT:

The authors generated and analyzed single-cell transcriptomic data of *Trypanosoma cruzi* using 10X Genomics technologies. This work represents one of the first single-cell transcriptomic datasets for this parasite and constitutes a valuable contribution to the community studying *T. cruzi*. Additionally, they provide an atlas that can be used to annotate life cycle stages and facilitate the annotation of UTRs. The work is of significant general interest; however, the manuscript requires substantial improvements in experimental design, and several important aspects of the data analysis need careful revision. Overall, we have observed that some paragraphs or sentences are incorrectly referenced, necessitating a thorough review. In the comments below, we will highlight some of these issues.

SAMPLES/DESIGN

To be considered an accurate single-cell atlas, other researchers should utilize a dataset as a reference. In this regard, the experimental design is critical. *Trypanosoma cruzi* shows significant variability depending on the clade/DTU used, which adds complexity to the analysis. Moreover, its life cycle is more intricate than the traditional classification, divided into four stages: epimastigotes, amastigotes, metacyclic trypomastigotes, and cell-derived trypomastigotes. During its intracellular cycle, *T. cruzi* transitions through additional intermediate and clearly defined stages during trypomastigogenesis, including intracellular epimastigotes. In this context, several study aspects need substantial modification or clarification.

- Strain: It is necessary to specify that it belongs to DTU I. While this has the advantage of representing strains circulating in humans and wildlife, it also means that the results may not be extrapolated to other DTUs.

- Epimastigotes (EP): The parasite concentration in the 2-day cultures from which the samples were taken must be explicitly stated.

- Metacyclic trypomastigotes (SE/MT): Line 764 states that the sample was obtained from a 10-day-old culture. It is essential to clarify that the parasites were previously subjected to starvation and to provide data on the final parasite concentration. Concerning metacyclic trypomastigotes, we strongly suggest adding one more step: DEAE column enrichment, which typically yields populations with >95% MT.

- ADT: It is essential to support this analysis with microscopy images of the three populations. Once trypomastigotes infect the host cell, two intracellular processes occur: amastigogenesis and trypomastigogenesis. Both processes involve intermediate and well-defined stages, with intracellular epimastigotes present before differentiating into trypomastigotes. Therefore, the term "ADT" should be revised to "CDT" (cell-derived trypomastigotes) to avoid the assumption that the preceding stage was amastigotes. Another critical point is the timing of CDT collection from the supernatant: these parasites should exit the cell no more than 12 hours earlier; otherwise, they begin transitioning into rounded forms known as extracellular amastigotes. Was this criterion taken into account? Can we ensure that the CDT population is greater than 90%? Again, microscopy images are essential.

- AMA: The progression of the intracellular cycle varies depending on the strain, cell type, and MOI. The authors used two MOIs (10 and 1.5) to obtain AMA samples, presumably to

maintain the 120-hour time point. However, this raises the question of which cellular form is actually being reported in the single-cell data. The authors rely on the work of Li et al., but that study used the Y strain belonging to DTU II, whose cycle timing differs from that of DTU I. In addition, intracellular epimastigotes precede the differentiation to trypomastigotes (see below). Therefore, returning to the fundamental issue, it is crucial to document the cellular forms used in sample collection with images. Otherwise, this study cannot be considered an atlas.

INTRODUCTION

This overly long introduction focuses too much on very general and well-known aspects of Chagas disease and its cycle. The life cycle explanation is overly detailed and could be summarized and modified to introduce the concept that, in addition to these four stages, there are additional and transitional stages (in particular, the intracellular epimastigotes constitute a defined stage), which also vary between clades or DTUs.

Articles that may contribute to revising the cycle:

- https://doi.org/10.1016/0014-4894(78)90159-5
- https://doi.org/10.1007/BF00931508
- https://doi.org/10.1006/expr.1999.4423
- https://doi.org/10.3390/pathogens13100866
- https://doi.org/10.1186/s13071-018-2664-4

Note that the last paper is reference 4 in the main manuscript. Although it is already cited, it is incorrectly referenced at the end of the sentence:

"Differentiation into non-motile, replicative amastigotes starts in parasitophorous vacuoles, followed by escape to the host cell cytosol, where differentiation is completed."

There are two errors here:

1. The cited article refers to metacyclogenesis, which means the cycle in the vector, while the authors discuss the intracellular cycle in mammals.
2. Amastigotes are not "non-motile" (https://doi.org/10.1128/mbio.03556-22).

RESULTS

- The reasoning for the authors' choice of pools for sequencing is unclear in the text and must be explained.

- We are deeply concerned about several aspects of the data analysis that require careful revision and reanalysis. As is well known, the genome of *T. cruzi* is highly repetitive; approximately 50% of the genome consists of repetitive sequences, including multigene families, repetitive elements, and transposable retroelements, among others. This genomic characteristic has posed significant challenges for scientists working with this parasite, who have extensively sought effective strategies to ensure accurate data interpretation and reliable results. A crucial part of the transcriptome analyses of *T. cruzi* is the treatment of multimapping reads. Excluding multimapping reads from the analysis oversimplifies biological reality. Evidence suggests that this is not a correct strategy. In this study, the authors conduct

expression analysis of multigene families for which, by their very nature, most of the reads generated by their transcripts are multimapping. This issue becomes even more critical, highlighting a significant aspect that requires review. The authors should re-evaluate and interpret the data, considering these factors.

- Line 79: the authors should not use abbreviations the first time they describe the samples (e.g., EP, SE, MT, AMA).

- Figure 1 illustrates the samples used in the study but does not present any results. Since the parasite's life cycle is already well-documented in the research community, creating a detailed schematic is unnecessary. Instead, a brief description of the sample collection process in the methods section would be sufficient. The figure should be moved to the supplementary material section.

- Figure 2. How do the authors explain the differences in the SE/MT replicates? In replicate 2, there appears to be a cluster of cells not present in replicate 1.

- UTR mapping and Figure 4. There is a missing citation and inclusion of an important study of UTR genome-wide annotation conducted on *T cruzi.* (https://doi.org/10.3389/fgene.2018.00671). The authors list several results that cannot be attributed to the only two figures they present in this section. They describe results from weak analyses (based on inspections of graphs but which are not shown). Further studies and quantifications to support the results described in the "UTR mapping" section are needed to substantiate these results. The authors must provide more evidence and information about the described results (lists of the "missing transcripts," methods, and criteria they used to say there is an unannotated transcript, the possibility that the "unannotated" genes are long ncRNAs).

- Figure 4. The authors list several results from which no merits can be derived from the figure. They describe results from weak analysis based on inspections of graphs, but they do not appear. Moreover, in T. cruzi, genes lack introns except for a few distinct exceptions; thus, referring to the coding portion of a gene as an exon is incorrect. Finally, as mentioned above, the sentence "Our analysis estimates that there are ~1,500 missing genes in the DM28c annotation" lacks foundation. What is the list? Or at least the location? We strongly recommend further argumentation and substantiation of these statements throughout the text.

- This is not the first time the transcription of both strands has been described in *T. cruzi*. This was very well documented in 2021 (https://doi.org/10.1371/journal.ppat.1009254). The authors should consider and include previous studies conducted on this parasite throughout the entire manuscript.

- Can these results be validated in other transcriptomes? To ensure consistency in the analysis, the scRNA-seq and bulk transcriptome results should be compared with the transcriptome published in 2023 (DOI: 10.1038/s41564-023-01483-y), which includes a deep sequencing analysis of three parasite stages from a DTU I strain (Dm28c used in this work for analysis). The comparison must focus mainly on marker genes to validate them as markers. Additionally, a comparison with the transcriptome from Li et al. should be performed to assess whether these markers apply to a strain from a different DTU (II in this case). With these comparisons, authors could establish whether the detected genes are definitively stage markers.

- Related to the previous point, it has been recently published a preprint (https://doi.org/10.1101/2025.01.14.633000) entitled "Single-cell RNA-seq reveals trans-sialidase-like superfamily gene expression heterogeneity in Trypanosoma cruzi populations." Authors perform single-cell RNA sequencing of T cruzi amastigotes and trypomastigotes and determine markers from each stage. It is essential to compare the markers. Note that the authors YES consider the multimapping reads in the referred preprint. It would be enriching to compare in the discussion the results of that other study in which there are some similar findings. It would be even more compelling to incorporate the results of an independent analysis that reaches similar conclusions.

- Line 862. The authors must specify the units to define the threshold, as "10" alone is meaningless.

- The authors describe only ONE gene for the amastigotes stages to indicate that the bulk RNA-seq validates the scRNA-seq atlas. However, the biology of *T. cruzi* is too complex to be reduced to discussing a single gene. We expect a more comprehensive analysis to enrich this section.

- Figure 3d: It would be interesting to know the results of the reverse analysis: how is the expression in single-cell RNA-seq of marker genes identified in bulk RNA-seq?

- Line 840: "The 3' UTRs annotations of the Trypanosoma cruzi Dm28c 2018 (73) were extended by…" Another mistake in the references. Reference 73 is a "Genome Announcement" from 2014, whereas Dm28c_2018, as its name suggests, was communicated in 2018.
-
- 211-213: "This analysis allowed us to identify highly specific marker genes for (…) of these in line with previously published results." Add the references for the previously published results.
